# The Epigenomic Landscape of Prokaryotes

**DOI:** 10.1371/journal.pgen.1005854

**Published:** 2016-02-12

**Authors:** Matthew J. Blow, Tyson A. Clark, Chris G. Daum, Adam M. Deutschbauer, Alexey Fomenkov, Roxanne Fries, Jeff Froula, Dongwan D. Kang, Rex R. Malmstrom, Richard D. Morgan, Janos Posfai, Kanwar Singh, Axel Visel, Kelly Wetmore, Zhiying Zhao, Edward M. Rubin, Jonas Korlach, Len A. Pennacchio, Richard J. Roberts

**Affiliations:** 1 Genomics Division, Lawrence Berkeley National Laboratory, Berkeley, California, United States of America; 2 U.S. Department of Energy Joint Genome Institute, Walnut Creek, California, United States of America; 3 Pacific Biosciences, Menlo Park, California, United States of America; 4 Physical Biosciences Division, Lawrence Berkeley National Laboratory, Berkeley, California, United States of America; 5 New England Biolabs, Ipswich, Massachusetts, United States of America; University of Minnesota, UNITED STATES

## Abstract

DNA methylation acts in concert with restriction enzymes to protect the integrity of prokaryotic genomes. Studies in a limited number of organisms suggest that methylation also contributes to prokaryotic genome regulation, but the prevalence and properties of such non-restriction-associated methylation systems remain poorly understood. Here, we used single molecule, real-time sequencing to map DNA modifications including m6A, m4C, and m5C across the genomes of 230 diverse bacterial and archaeal species. We observed DNA methylation in nearly all (93%) organisms examined, and identified a total of 834 distinct reproducibly methylated motifs. This data enabled annotation of the DNA binding specificities of 620 DNA Methyltransferases (MTases), doubling known specificities for previously hard to study Type I, IIG and III MTases, and revealing their extraordinary diversity. Strikingly, 48% of organisms harbor active Type II MTases with no apparent cognate restriction enzyme. These active ‘orphan’ MTases are present in diverse bacterial and archaeal phyla and show motif specificities and methylation patterns consistent with functions in gene regulation and DNA replication. Our results reveal the pervasive presence of DNA methylation throughout the prokaryotic kingdoms, as well as the diversity of sequence specificities and potential functions of DNA methylation systems.

## Author Summary

DNA methylation is a chemical modification of DNA present in many prokaryotic genomes. The best-known role of DNA methylation is as a component of restriction-modification systems. In these systems, restriction enzymes target foreign DNA for cleavage, while DNA methylation protects the host genome from destruction. Studies in a handful of organisms show that DNA methylation may also act independently of restriction systems and function in genome regulation. However, a lack of technologies has limited the study of DNA methylation to a small number of organisms, and the broader patterns and functions of DNA methylation remain unknown. Here we use SMRT-sequencing to determine the genome wide DNA methylation patterns of more than 200 diverse bacteria and archaea. We show that DNA methylation is pervasive and present in more than 90% of studied organisms. Analysis of this data enabled annotation of the specific DNA binding sites of more than 600 restriction systems, revealing their extraordinary diversity. Strikingly, we observed widespread DNA methylation in the absence of restriction systems. Analyses of these patterns reveal that they are conserved through evolution, and likely function in genome regulation. Thus DNA methylation may play a far wider function in prokaryotic genome biology than was previously supposed.

## Introduction

DNA methylation has widespread roles in the regulation of eukaryotic genomes [1–3], but the extent to which similar processes exist in prokaryotes is unknown. Methylated DNA is found in the genomes of bacteria and archaea in the forms of 6-methyladenosine (m6A), 4-methylcytosine (m4C), and 5-methylcytosine (m5C)[4], and is the product of DNA methyltransferase (MTase) enzymes [5]. MTases are often a component of restriction-modification (RM) systems [6], but have also been implicated in DNA mismatch repair [7] and other epigenetic regulatory phenomena [8]. While MTase genes are present in the genomes of many prokaryotes, the overall abundance and patterns of prokaryotic DNA methylation, and the functional diversity of MTases remains largely unknown.

RM systems play a central role in prokaryotic defense, and their constituent enzymes are foundational tools in modern molecular biology [6]. RM systems comprise a restriction endonuclease (REase) and a MTase with the same DNA binding specificity. The REase degrades DNA from viruses and other exogenous sources, while the cognate MTase methylates potential REase target sites in the host genome and thus protects them from cleavage. RM systems are classified into four main types [5, 6, 9, 10]. Type I RM systems are complex, multi-subunit systems composed of separate REase and MTase subunits, and a common DNA recognition specificity (S) subunit [11]. The S subunit in combination with two MTase subunits methylates DNA, while the S subunit in combination with two MTase subunits and two REase subunits results in restriction. Type I RM systems recognize bi-partite motifs (e.g. CAGNNNNNTCA), and cleave at large distances (up to several kb) from their binding site. Type II RM systems comprise separate REase and MTase enzymes, which are expected to show identical DNA binding specificity [12]. They bind short, mostly palindromic, motifs (e.g. GATC), and cleave DNA within or close to the recognition site. Exceptions are the Type IIG RM systems that are single chain polypeptides containing both DNA restriction and methylation activities, bind short non-palindromic sequences (e.g. GCCCAG), and cleave DNA outside of the DNA binding site [12]. In Type III systems the MTase alone contains a DNA binding specificity domain and forms a complex with the REase in order to restrict [13]. They bind short non-palindromic motifs (e.g. CGAAT) and cut outside of the DNA binding site. Finally, Type IV RM systems cut modified DNA and do not have a MTase component [14].

Knowledge of the binding specificities of RM systems is critical to understanding their biological functions. Traditional approaches to determine RM system specificities rely on patterns of DNA cleavage by REases, a strategy that limits discovery largely to Type II RM systems where the REase binds and cleaves DNA at the same location [5]. Owing to this limitation, while the DNA binding specificities of several thousand Type II RM systems are known, typically fewer than 100 of each of the other types of RM system are known [5]. For Type I, IIG and III systems that cut outside of the RM binding site, a more recent alternative approach is to take advantage of the identical motif specificities of methylation and restriction. In these cases, determination of the sequences methylated by the MTase can directly reveal the recognition sequence of the accompanying REase, as recently demonstrated for individual RM systems [15–21].

Beyond RM systems, MTases can also be involved in prokaryotic genome regulation [8, 22]. These enzymes are typically observed as ‘orphan’ MTases that are found encoded in prokaryotic genomes in the absence of genes encoding a cognate restriction enzyme [23]. Examples include the Dam MTases that regulate DNA replication timing and gene expression of Gammaproteobacteria [24] and the CcrM MTases that regulate cell cycle progression of Alphaproteobacteria [19, 25]. While genome-wide methylation analysis of individual genomes can in principle identify regulatory MTases and provide insight into the associated regulatory DNA methylation system [17, 18, 20, 21, 26, 27], in the absence of systematic mapping efforts it has remained unclear how common such mechanisms are in prokaryotes. It is unknown whether the MTases associated with RM systems can also play a regulatory role.

MTase-encoding genes are present in the majority of bacterial and archaeal genomes, suggesting that DNA methylation may be similarly abundant. Bisulfite sequencing has enabled genome-wide surveys of 5mC methylation [28, 29], but a historic absence of tools for studying m6A and m4C modifications that predominate in prokaryotic DNA[30] has precluded more comprehensive studies. It has recently been demonstrated that kinetic analysis of single molecule, real-time (SMRT) sequencing data can directly detect many types of DNA modification [4, 31, 32]. While this approach is only modestly sensitive to m5C methylation, it is capable of detecting both m6A and m4C highly with a high degree of accuracy and sensitivity. The application of SMRT sequencing to a small number of prokaryotes enabled the identification of methylated motifs, and annotation of the respective MTases [15–21].

In the present study, we systematically use SMRT sequencing to uncover the patterns of DNA methylation across a large panel of more than 200 diverse bacterial and archaeal genomes to provide an overview of the epigenomic landscape of prokaryotes. In so doing we reveal the ubiquity of DNA methylation, and annotate DNA binding specificities for hundreds of MTases belonging to previously intractable types of RM systems. Furthermore, we demonstrate that a large proportion of the ‘orphan’ MTase genes encoded in prokaryotic genomes are active under normal conditions and produce patterns of DNA methylation that are consistent with gene regulatory functions. Our findings provide evidence for the pervasiveness and potentially diverse functions of DNA methylation in prokaryotic genomes.

## Results

### Genome-Wide Methylation Patterns in 230 Diverse Prokaryotes

**Fig 1:**
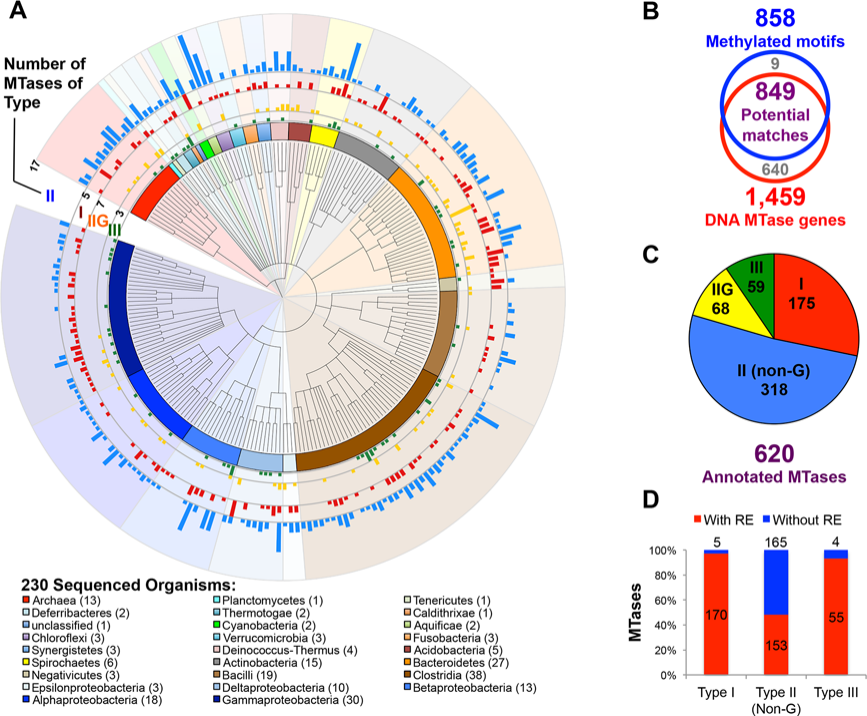
Methylomes of 230 prokaryotes. **A)** Phylogenetic tree of 230 sequenced organisms. Outer bars indicate the number and types of active MTases detected per genome **B)** Number of methylated motifs and MTase genes identified across all 230 organisms. **C)** Breakdown of 583 annotated DNA MTases by type. **D)** Proportion of annotated DNA MTases with and without cognate restriction enzymes. For Type IIG systems, MTase and restriction activities are encoded in the same peptide.

To explore the locations and potential functions of DNA methylation across prokaryotes, we selected 230 organisms for study, including 217 bacterial and 13 archaeal species, spanning 19 different phyla and 37 different classes (**Fig 1A, S1 Table**). These organisms were selected primarily based on their phylogenetic diversity to enable a comprehensive survey of bacterial methylation systems and maximize the chances for discovery of novel systems. For each organism, we isolated genomic DNA, and performed deep single molecule, real-time (SMRT) sequencing. We obtained on average 130-fold read coverage per organism, resulting in a combined dataset size of more than 79 million single-molecule reads and 105 Gb across all sequenced genomes. We aligned all SMRT sequences to the respective reference genomes, and used kinetic data analysis to identify the locations and probable types (m6A, m4C, m5C) of high-confidence base modifications in each sequenced genome (see **Methods**). We then identified sequence motifs that were recurrently methylated in each genome (**Methods**). The results of these analyses were genome-wide basepair-resolution methylation maps for each organism examined, as well as a set of modified motifs for each genome, where each motif represents the likely binding specificity of a DNA MTase.

In total we identified 858 methylated motifs, with DNA modifications detected from 215 / 230 organisms (93%), and across all sequenced phyla (**Fig 1A**). On average, we observed 3 methylated motifs per organism, with a maximum of 19 in *Neisseria gonorrhoeae*. Among modified motifs, the predominant base modification type detected was m6A (75%), with m4C and m5C accounting for 20% and 5%, respectively (**S1 Fig**). The large number of m6A methylated motifs is consistent with the frequent occurrence of this modification type in the database of known MTase specificities [5], and the ease with which this modification type is detected by SMRT sequencing. In contrast, the low frequency of m5C methylated motifs is an underestimate of the true number of such motifs across these genomes due to the lower sensitivity of SMRT sequencing to this modification type (**S2 Fig**)[16]. The fifteen organisms without detectable methylation are from across the sampled taxa, with no obvious shared characteristics. In 8/15 cases, their genomes lack predicted MTase genes (but harbor methyl-directed restriction enzymes), while in other cases MTases are present but were not detectably active by SMRT sequencing (**S2 Table**). In summary, these data reveal that DNA methylation is widespread across prokaryotes, and provide a valuable resource for exploring the specificities and functions of the MTases present in these genomes.

### Systematic Annotation of DNA Methyltransferase Specificities

To identify the individual MTases responsible for each methylated motif, we performed large-scale annotation of MTase binding specificities across the studied genomes. Using an integrative RM-system gene annotation pipeline (**Methods**), we identified 1,459 candidate MTase genes across the 230 genomes, and classified them according to RM-system type (panel A in **S3 Fig**). We then similarly classified the 858 detected motifs according to the type of MTase system to which they likely belong (panel B in **S3 Fig**). Comparison of the types of methylated motifs and MTase genes within the same organism enabled us to make initial predictions of the MTase enzyme responsible for each observed methylated motif (**Fig 1B**). For nearly all detected methylated motifs (849, 99%), we identified at least one candidate MTase in the same genome predicted to be capable of producing the modification. In contrast, there were many (640, 44%) candidate MTase genes for which no potential modification activity was detected. Of these 227 are MTases that are predicted to produce m5C modifications that are difficult to detect by SMRT sequencing. Other cases may be MTases that are inactive due to genetic drift, mis-identified enzymes that target RNA or protein rather than DNA, or genes that are not expressed, as frequently occurs when MTases are located on prophages.

In 620 cases, we were able to unambiguously match a single candidate MTase to a motif of the same type in the same genome (**Fig 1C**), thus generating a set of high confidence annotations of MTase specificities (**S3 Table** and **S4 Table**). The remaining unmatched motifs are due to several candidate MTases being present in the same genome, with insufficient evidence to make an unambiguous assignment.

For almost all Type I and III MTase gene predictions, a cognate REase was identified in the same genomic region, suggesting that these constitute intact RM systems, and enabling the systematic annotation of restriction specificities (**Fig 1D, S1 Text**). In contrast, restriction enzyme candidates could not be identified for over half (165/318) of the Type II MTases that are present (**Fig 1D**). This is consistent with the previous observation that Type II MTases frequently occur as orphans in bacterial genomes [23]. While we cannot exclude the possibility that some novel REase genes were not identified due to sequence divergence, these 165 orphan Type II MTases represent a large group of MTases with likely non-RM functions.

### Expanding the Repertoire of RM System Specificities

**Fig 2:**
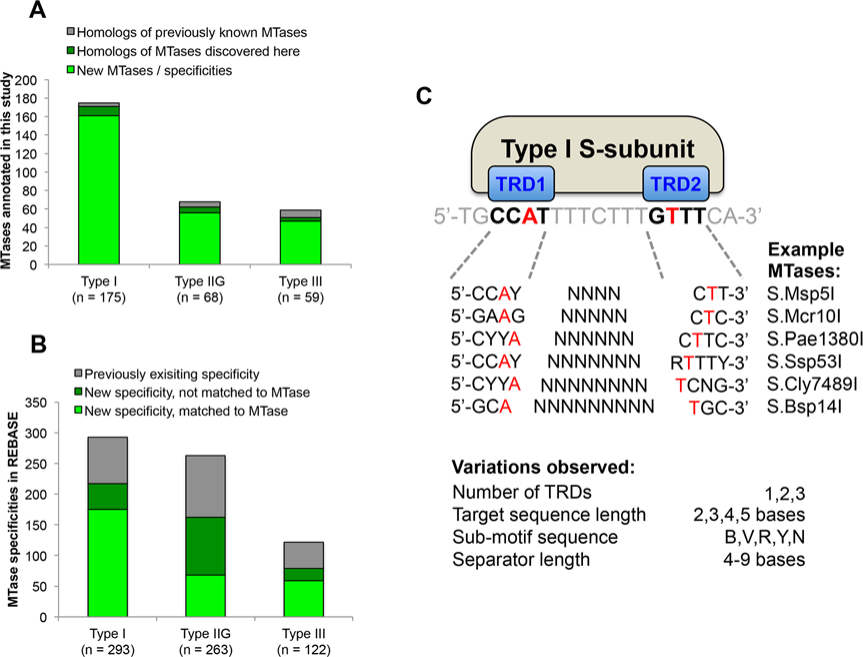
Novelty and diversity of methylome data. **A)** Novelty of MTases annotated in this study. The 583 newly annotated MTases were compared against all existing annotated MTases in the REBASE database [5], and classed as novel or homologous to existing MTases on the basis of their sequence specificities. **B)** Impact of this study on the diversity of known MTase specificities. ‘Previously existing’ refers to all specificities in the REBASE database prior to this study. New specificity refers to methylated motifs identified in this study. **C)** Diversity of DNA recognition by Type I RM systems. Type I specificity subunits recognize bipartite motifs. In this data, all aspects of this architecture are found to vary. Red bases indicate the positions of 6mA methylation (A), or their reverse complement (T). Sub-motif sequence variation shows the IUPAC codes for ambiguous nucleotides found within the bipartite DNA sequences.

Comparison with known RM systems [5] indicates that our systematic analysis identified 148 RM systems with previously undescribed sequence determinants, substantially expanding the repertoire of available specificities. The discovery rate of novel enzyme specificities was particularly large for Type I, IIG, and III RM systems that have been historically difficult to study using conventional approaches (**Fig 2A**). For example, 92% (161/175) of annotated Type I system specificities identified in our study were novel. In addition, among the Type I motifs that could not be matched to genes the majority were new specificities not seen previously. As a result, our analysis increases the number of known Type I system specificities almost four-fold (from 76 to 293, **Fig 2B**). Our data also reveals the extraordinary diversity of modes of DNA recognition by Type I RM systems, with variation observed in all aspects of the DNA recognition architecture (**Fig 2C**).

We also identified a substantial number of novel recognition specificities by Type IIG and Type III MTases. Among Type IIG RM systems annotated, 82% (56/68) were novel, while the same was true for 79% (47/59) of the Type III specificities (**Fig 2A**). Unmatched motifs in these categories cannot always be unambiguously attributed as being from a Type IIG or Type III enzyme because both lead to characteristic single-strand methylation. Preliminarily we have considered short recognition sequence of 4 or 5 bases to most likely belong to the Type III family, while the longer recognition sequences of 6 or more base pairs are considered as Type IIG. Overall, the number of observed specificities across these Types of restriction system increased 2.7-fold (from 144 to 385) as a result of our study.

### Novel Protective Modifications

Previously, protection against Type I restriction enzymes was always found to be mediated by m6A modification [11]. In this study, we find examples of protection by m4C (M.Dac11109IV in *Desulfobacca acetoxidans* and M1.Mma5219I in *Methanohalophilus mahii*, **S3 Table**). Similar results have been obtained from other recent studies [5], and several of these systems have now been experimentally verified (Morgan et al. personal communication). Interestingly, when this happens there are two MTase genes associated with the system, one of which appears responsible for m6A methylation and the other for m4C methylation. In these cases the bipartite recognition sequence of the Type I S subunit has only G and C residues in one of the target recognition domains, which explains why m6A cannot be used to protect both halves. There are many homologs elsewhere in REBASE of systems like this, but often the specificity is unknown [5]. A similar situation has also been found for some Type III MTases where occasionally m4C is found as the protective modification both in some of the systems identified here as well as others [5].

### New Families of Type IIG-Like Restriction Systems

Type IIG systems are defined by the presence of a single target recognition domain (TRD) for the entire RM system. They typically consist of a single polypeptide containing both the endonuclease domain and m6A MTase, as in the prototypical enzyme MmeI [33] **(S4A Fig)**. Here, we identified 76 novel Type IIG-like systems, many of which were atypical in terms of gene order, presence or absence of a DNA translocase, and differences in linkage between the endonuclease and MTase domains (**S3 Table** and panels B-E in **S4 Fig**). For example, we identified several different systems in which one peptide contains an MmeI family MTase/TRD, but in which the endonuclease is encoded on a separate peptide (AchA6III and OspHL35III, panels B and C in **S4 Fig**). Other examples such as CalB3II (panel D in **S4 Fig**) are new examples of BREX-like systems [34]. These systems use the specific methylation of the MTase protein to distinguish self from non-self in phage restriction, but appear to accomplish restriction without generating DNA cleavage. Finally, we observe novel systems that are unrelated to MmeI or BREX. For example, MexAMORF1192P is a four-protein system of two translocase proteins and separate MTase-TRD and endonuclease proteins (panel E in **S4 Fig**). These analyses highlight the value of SMRT-sequencing in annotating novel RM systems. The examples we describe represent just a portion of the wide diversity of Type IIG-like systems that evolve from various permutations of endonuclease, MTase and translocase domains with a single DNA recognition module. The preliminary annotations of Type IIG-like MTases from this study can be propagated across many orthologs and will enable their further characterization and systematic classification.

### An Unusual Type II RM System

While Type II RM systems represent historically the best-studied class of RM systems, our systematic survey identified a substantial number of new Type II RM systems, some of which have unusual properties. For example, all Type II RM systems described to date are characterized by close genomic proximity of the genes encoding the REase and the MTase, respectively [5]. We observed one pair of adjacent MTases M1.Csp12AI and M2.Csp12AI in *Clostridium sp*. *12(A)* that were very similar to the m6A-MTase M.FokI from *Flavobacterium okeanokoites*. However, in *Clostridium sp*. *12(A)* the gene encoding the corresponding FokI-like restriction enzyme was not found in the immediate vicinity of M1/M2.Csp12AI, but at a genomic location 1.2 megabase pairs (Mb) away. All three genes were tested for activity by cloning. While M2.Csp12AI could be cloned alone, it was only possible to clone the M1.Csp12AI gene in the presence of M2.Csp12AI. In both cases, just as in the genome, both MTases were shown to be fully functional by PacBio sequencing of DNA (**S5 Fig**). To exclude the possibility that the large apparent distance resulted from an incorrect genome assembly, we confirmed by PCR that the distance between the REase gene and the two MTase genes is at least 36 kb (**S6 Fig**). These results indicate that, unlike all previously described Type II RM systems, there are Type II RM systems in which the REase and MTase genes are located at distant sites on the chromosome.

### Families of Active ‘Orphan’ MTases Are Conserved across Diverse Prokaryotic Phyla

**Fig 3:**
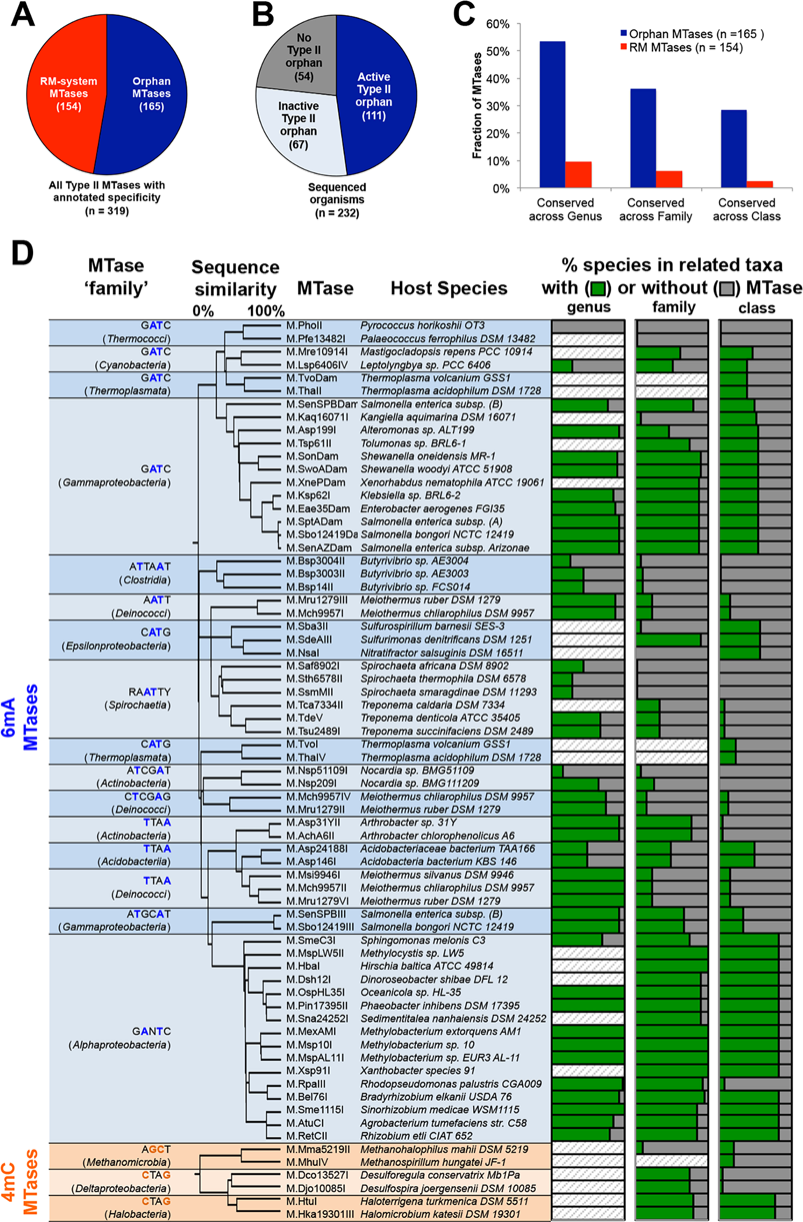
Identification and classification of ‘Orphan’ Type II MTases. **A)** Proportion of Type II MTases encoded with (RM-system) or without (‘orphan’) a cognate restriction system. **B)** Proportion of sequenced organisms with orphan Type II MTases. **C)** Comparison of evolutionary conservation properties of Type II MTases. An MTase is considered conserved if orthologs are present in >50% species in the respective taxonomic class. **D)** Table of orphan MTase families identified based on protein sequence clustering (**Methods** and **S2 Fig**). Tree is based on agglomerative clustering of protein sequences. Bar charts represents fraction of related species with (green) or without (grey) a copy of the orphan MTase gene (diagonal lines, < 5 sequenced genomes).

Our systematic survey identified 165 candidate ‘orphan’ Type II MTases (**Fig 3A, S3 Table** and **S4 Table, Methods**). These MTases are found in isolation, i.e. in the absence of corresponding restriction enzymes, but nonetheless actively methylate specific sites in the genome. This feature raises the possibility that these MTases are involved in non-RM-functions, such as gene regulation. Orphan MTases are widely distributed among prokaryotes with at least one example in 111 (48%) organisms and 15/20 different phyla included in this study (**Fig 3B)**.

To explore the properties and potential functions of orphan MTases in more detail, we first examined the phylogenetic conservation of orphan and RM system MTases. We determined the presence or absence of each MTase among all sequenced species related to the host organism at the genus, family or class level, and with an available reference genome sequence (**Methods).** We considered MTases to be conserved if present in at least 50% of species within the respective taxonomic group (**Fig 3C**). Overall, orphan MTases are far more likely to be evolutionarily conserved than RM system-associated MTases. For example, the majority of orphan MTases (57%) are conserved at the genus level, while the same is true for only 9% of RM system MTases. A similar contrast between orphan and RM MTases is observed at the level of family and class (**Fig 3C**). These results are consistent with a greater degree of conservation of orphan MTases compared with RM MTases [23], and suggest that orphan MTases have functional roles distinct from host protection.

We next performed protein sequence similarity-based clustering to identify candidate novel families of related orphan MTases. We generated initial protein clusters from all 260 Type II MTases in our study (**S7 Fig** and **S8 Fig**), then extracted sub-clusters of orphan MTases from taxonomically related host organisms and with identical motif recognition sequences (**Methods**). These analyses resulted in 19 orphan MTase families accounting for 107 / 165 orphan MTases in our study (**Fig 3D)**. The remaining 58 MTases are ‘singletons’ with no ortholog in any other genome in our dataset.

The two most highly represented orphan MTase families in our study are the known regulatory orphan Dam MTases in Gammaproteobacteria, and CcrM MTases in Alphaproteobacteria, reflecting our large sampling of organisms from these taxa. Of the remaining 17 candidate families, 3 are apparent homologs of Dam MTases in Cyanobacteria and two archaeal classes, respectively. The other 12 families are novel orphan MTases of unknown function and are found in diverse prokaryotes including both bacteria and archaea. The most highly represented orphan MTase family methylates the motif 5’-RA^**m6**^**A**TTY-3’ (T indicates that the A on the complementary strand is modified) in all six Spirochaetaceae sequenced as part of this study. This motif and orphan MTase had previously been observed in *Campylobacter jejuni* [16].

In many cases, novel orphan MTase families are widely conserved in genomes beyond those included in our study. For example, the gene for the orphan MTase targeting 5’-TTA ^**m6**^**A**-3’ in two Arthrobacter species in our study is present in 39 / 42 (93%) of all sequenced genomes from the genus Arthrobacter. Similarly the orphan MTase targeting 5’-^**m4**^**C**ATG-3’ in two Haloarchaeal species in our study is present in 121 / 156 (78%) of all sequenced genomes from the class Haloarchaea (**Fig 3D**).

In summary, these analyses reveal the presence of several novel evolutionarily conserved families of orphan MTases of unknown function.

### Orphan Type II MTases Are Associated with Unmethylated Sites in Gene Regulatory Regions

We hypothesize that some of the newly discovered orphan MTases function similarly to the known regulatory orphan MTases Dam and CcrM, i.e. that they regulate gene expression through the presence or absence of methylation in regulatory sequences. Alternatively their function may be to regulate DNA replication, through clusters of motifs in regions of the genome associated with DNA replication control [35]. To explore these possibilities in more detail, we searched our methylome data for signatures consistent with such functions.

It has previously been shown that a subset of target sites of the *E*. *coli* regulatory MTase Dam is completely unmethylated [36–38]. These unmethylated sites are the consequence of the competing activities of *Dam* MTase and regulatory proteins, and the presence or absence of methylation at these sites has a demonstrated impact on gene expression [39, 40]. We therefore asked if we could recapitulate these findings for Dam MTases in our dataset, and if similar patterns are associated with novel orphan MTases.

**Fig 4:**
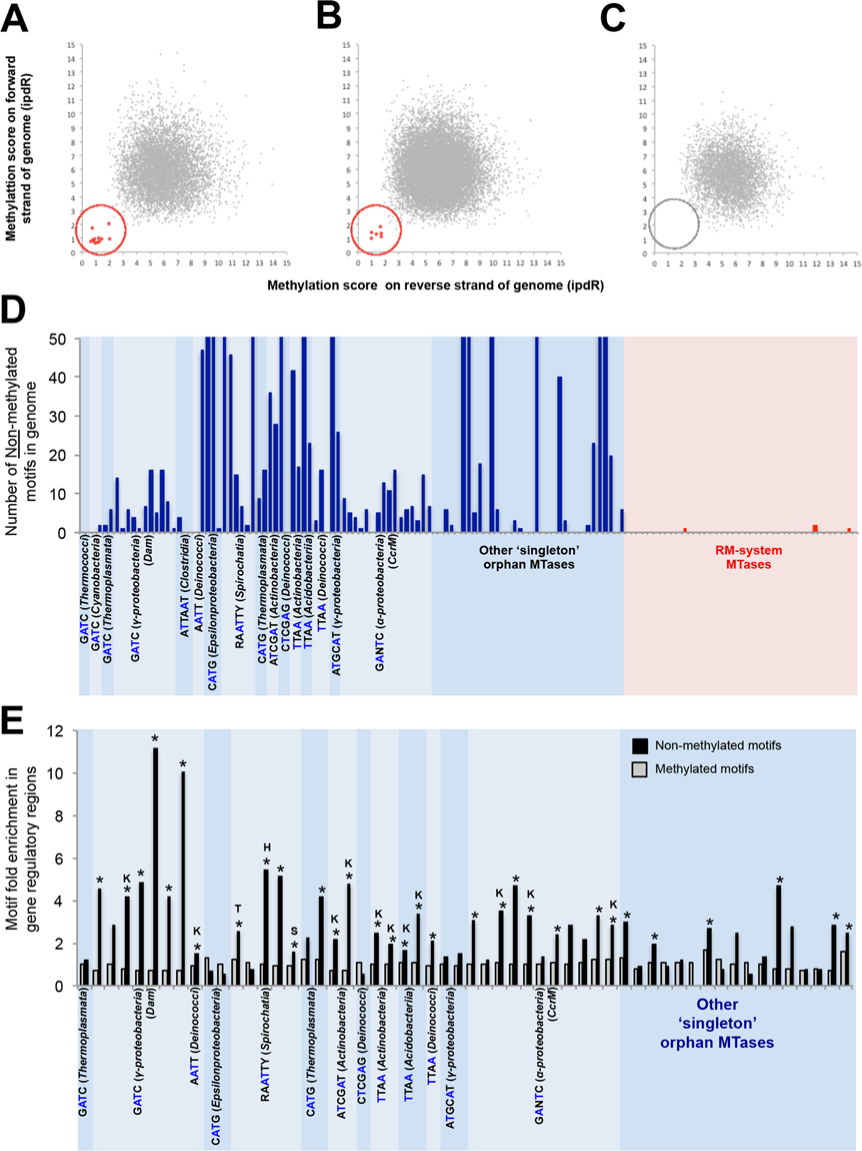
Orphan MTases are associated with unmethylated sites in the gene regulatory regions. **A)** Scatter plot of DNA modification scores on forward and reverse strands of each Dam MTase target motif (GATC) in the *E*. *coli* genome. The ipdR (inter-pulse duration ratio) is the primary metric in DNA modification detection, and corresponds to the time delay in incorporation of successive bases in a sample versus an unmodified control. This plot reveals a distinct set of sites that is unmethylated on both strands of the genome (highlighted in red). **B)** Scatter plot of DNA methylation scores at Dam target motif sites in *Salmonella bongorii* reveals a similar set of unmethylated sites. **C)** Scatter plot of DNA methylation scores GATC-specific RM-system MTase in *Clostridium thermocellum*. In this case all sites in the genome are methylated. **D)** Systematic analysis of the number of unmethylated motifs (on both strands of the genome) associated with orphan MTases (blue panel), and RM-system MTase (red panel). Orphan MTase names and gene orders correspond to **Fig 3**. **E)** Fold enrichment of all motifs (grey bars) and unmethylated motifs (black bars) in gene regulatory regions. * = significantly enriched (p <0.01, Fishers exact). Letters indicate enrichment at specific functional categories of genes based on COG category analysis. K = transcription, T = signal transduction, H = coenzyme metabolism.

In the *E*. *coli* data from this study, the vast majority (17,544/17,562, 99.9%) of 5’-G ^**m6**^**A**TC-3’ motifs are fully methylated on both strands of the genome. However, a distinct set of 18 5’-G ^**m6**^**A**TC-3’ motifs is unmethylated on both strands of the genome (**Fig 4A**). These unmethylated sites include six GATC positions in upstream regulatory regions of *agn43* genes that are known to be regulatory targets of Dam methylation [39]. Unmethylated sites are also detected in association with the *dam* orphan MTase gene of *Salmonella bongorii*, (**Fig 4B**, and **S5 Table**). In contrast, unmethylated sites are absent from the genome of *Clostridium thermocellum*, a bacterium harboring a 5’-G ^**m6**^**A**TC-3’ specific MTase that is part of an RM system (**Fig 4C**). These results suggest that the presence of small subsets of reproducibly unmethylated recognition motifs across the genome may be a distinctive signature of orphan MTases.

We extended this analysis to all m6A orphan and RM-system associated MTases in our dataset with sufficient SMRT sequencing coverage for confident detection of unmethylated sites (**Methods**). We observed widespread occurrence of unmethylated sites in association with Dam MTases across Gammaproteobacteria, as well as with the regulatory CcrM orphan MTases in Alphaproteobacteria (consistent with recent observations of unmethylated sites in *Caulobacter* [21]). Strikingly, we also observed unmethylated sites in association with at least one MTase for the majority (13/16) of novel orphan MTase families, as well as with over half of ‘singleton’ orphan MTases (**Fig 4D, S9 Fig** and **S5 Table**). In contrast, MTases of restriction systems are almost always associated with complete modification of their genomes, with only four apparently unmethylated sites observed across 41 RM MTases (**Fig 4D)**, and consistent with a role in protecting the genome from the cognate restriction enzyme. On further inspection, all four apparent unmethylated RM MTase sites have modification scores at the borderline of detection, and likely represent the background false-positive rate of detection of unmethylated sites. Overall these analyses confirm that unmethylated motifs are a common signature of novel orphan MTases, and may represent novel regulatory sites in the genome.

In known cases of gene regulation by orphan MTases, functionally relevant motif sites are located in regulatory sequences upstream of genes and are unmethylated in some or all of the population [39, 41]. We therefore asked whether the target motifs of the orphan MTases identified in this study are similarly associated with gene regulatory regions (**Fig 4E**). In general, orphan MTase motifs (irrespective of their methylation state) are not significantly enriched at gene regulatory regions (defined as 100bp upstream of CDS start to 50bp downstream of CDS start, **Fig 4E, grey bars**). However, two-thirds of orphan MTases are associated with a significant enrichment of unmethylated motifs in gene regulatory regions (**Fig 4E, black bars**). Furthermore, unmethylated motifs are especially enriched in the promoters of genes of related function, most notably transcriptional regulators (**Fig 4E**). For example, in *Nocardia sp* BMG111209, unmethylated 5’-ATCG^**m6**^**A**T-3’ motifs are 5-fold enriched in gene regulatory regions, compared with fully methylated motifs (17/28 (61%), compared to 13% by chance). This enrichment increases to more than 20-fold for unmethylated sites upstream of transcriptional regulators (7/28 (25%) unmethylated motifs compared with only 1.2% methylated motifs, *p* < 0.01). Finally, at least in the case of *dam* methylases in gammaproteobacteria, unmethylated motifs overlap predicted transcription factor binding sites significantly more frequently than do methylated motifs (**S10 Fig** and **S5 Table**).

Overall, these results demonstrate a substantial enrichment of unmethylated motifs in regulatory regions of the genome. Since this enrichment is not merely a consequence of an elevated density of motifs in these regions, it may instead reflect the involvement of these sites in regulatory processes. The patterns of novel orphan MTases (including ‘singleton’ MTases) resemble those of the known MTases Dam and CcrM, further supporting the possibility that they may have shared functions in the epigenetic control of gene expression.

### Identification of Putative Novel Targets of Orphan MTase Regulation

While our analyses are generally consistent with a role for orphan DNA MTases in gene regulation, it is unclear which unmethylated sites represent targets of regulation. Indeed, previous studies of unmethylated sites have shown that while some sites are important in regulating gene expression, others may represent inconsequential blocking of DNA methylation by tightly bound transcription factors [41, 42]. We therefore sought to prioritize our data to identify individual cases of putative regulation.

**Fig 5:**
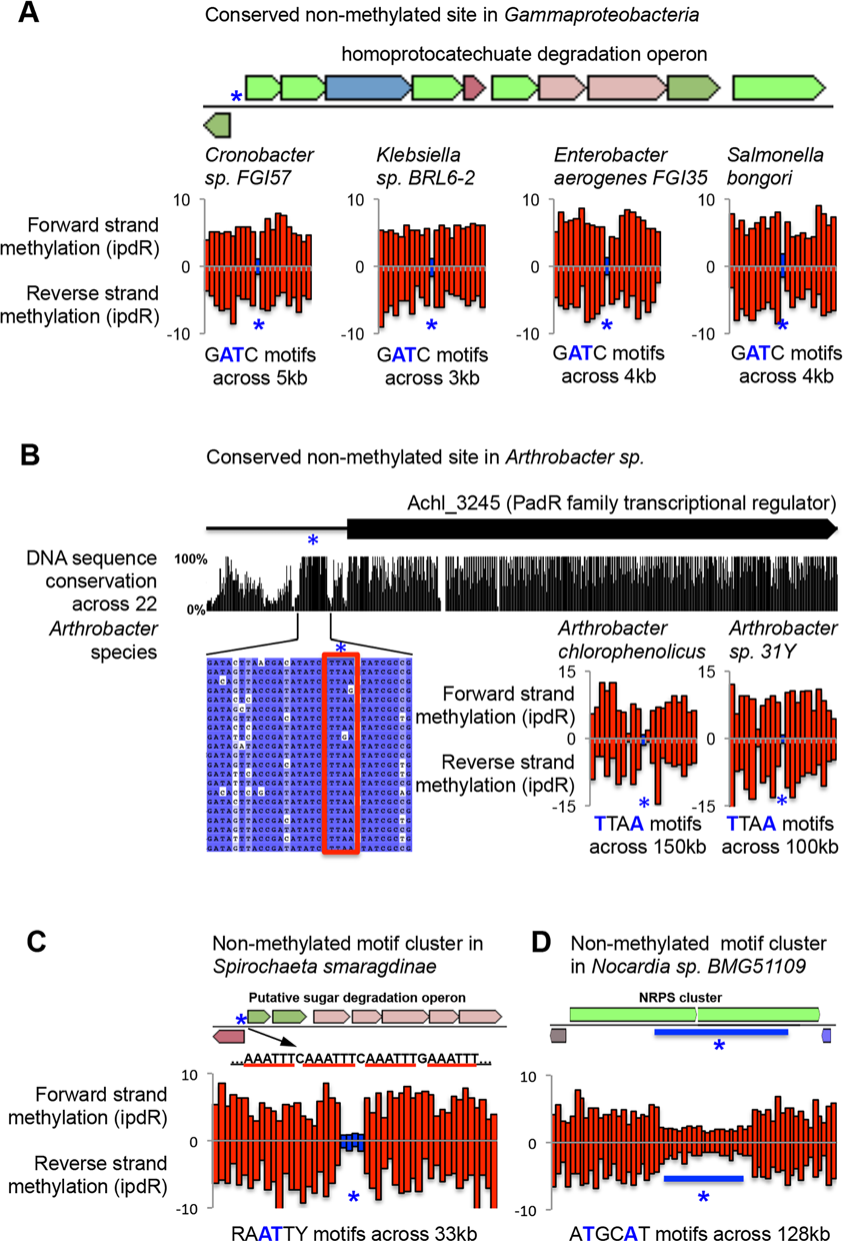
Examples of candidate regulatory unmethylated sites. In all panels bar charts show the extent of DNA methylation (inter-pulse duration ratio) at the candidate regulatory unmethylated site (blue) and, for comparison, at the ten immediately flanking upstream and downstream motif instances (red). The sequence interval covered in each chart varies due to the density of motifs across the respective genomes. **A)** Unmethylated sites are present upstream of the Hpa operon in four *Enterobacteria* species. In 3 cases, the unmethylated site is at the orthologous GATC, in *S*. *bongori*, the unmethylated site is located ~100bp upstream of the conserved sites. **B)** Conserved unmethylated site upstream of a PadR transcriptional regulator in *Arthrobacter* species. **C)** Cluster of unmethylated sites upstream of transcriptional regulator and sugar degradation operon in *Spirochaeta smaragdinae*. **D)** Cluster of non- or weakly-methylated sites throughout a non-ribosomal peptide synthase operon.

We first searched for unmethylated motifs at the same genomic location across multiple related organisms. This analysis revealed 14 candidate regulatory sites across 5 different orphan MTases (**Table 1**). Among conserved unmethylated sites is one upstream of the glucitol/sorbitol specific PTS system (gut locus). This site was previously identified in *E*. *coli*, and appeared to have no impact of gene regulation [41], nevertheless the absence of methylation at this locus is strikingly well conserved across the Enterobacteria in our study (**S11 Fig**). We identified eight other Dam motifs at conserved locations and unmethylated in at least two Gammaproteobacteria (**Table 1** and **Fig 5A**). We also identified conserved sites in association with three novel orphan MTases. For example, we identify conserved unmethylated sites upstream of a PadR family transcriptional regulator in both *Arthrobacter* species, and show that the motif in question is extensively conserved across the *Arthrobacter* genus (**Fig 5B**).

**Table 1:** Evolutionarily conserved unmethylated motifs. Conserved unmethylated sites were identified based on reciprocal best blast hits of flanking genes sequences between the respective genomes. Bold and underlined characters indicate methylated bases. Underlined characters indicate reverse complement of methylated bases.

MTase family	Location of conserved unmethylated site	Number of organisms with conserved unmethylated motif
**GATC (Gammaproteobacteria)**	Upstream of PTS system D-sorbitol-specific IIC component	6
	Upstream of homoprotocatechuate degradation operon regulator, HpaR	4
	Upstream of Sugar kinases, ribokinase family	3
	Upstream of putative diguanylate cyclase	2
	Upstream of putative TetR-family regulatory protein	2
	Upstream of phage repressor protein cI	2
	Upstream of transcriptional regulator, GntR family	2
	Upstream of putative zinc-type alcohol dehydrogenase	2
	Upstream of hemin receptor protein	2
**RAATTY (Spirochaeta)**	Upstream of transcriptional regulator, LysR family	2
**ATCGAT (Nocardiaceae)**	Upstream of transcriptional regulator, GntR family	2
**TTAA (Arthrobacter)**	Upstream of transcriptional regulator, PadR family	2
	Upstream of transcriptional regulator, ArsR family	2
**GANTC (Alphaproteobacteria)**	Upstream of transcriptional regulator, MarR family	2

We next searched for the presence of unusual clusters of adjacent unmethylated motifs in related regions of the gene regulatory region, and identified seven potential regulatory regions across six orphan MTases (**Table 2**). Among these regions are known regulatory sites upstream of the *agn43* locus in *E*. *coli* [39], supporting the validity of this approach for finding true regulatory sites. We also identified a novel cluster of unmethylated Dam target sites upstream of a TonB-dependent receptor and putative iron uptake operon in *E*. *coli*. In addition, we identify clusters of unmethylated sites in association with three novel orphan MTases. These include a cluster of sites upstream of a GntR family transcriptional regulator and putative sugar utilization operon in *Spirochaeta smaragdinae* (**Fig 5C**). More unusually we observe an extended region of reduced methylation along the entire length of an RPS synthesis gene in *Nocardia* sp. BMG51109 (**Fig 5D**).

**Table 2:** Clusters of multiple adjacent unmethylated motifs. (* = Unmethylated motif clusters and putative target genes each occur in two copies in the respective genomes). Bold and underlined characters indicate methylated bases. Underlined characters indicate reverse complement of methylated bases.

Orphan MTase	Location of unmethylated motif cluster	Number of adjacent unmethylated sites
**ATCGAT (*Nocardiaceae*)**	Throughout non-ribosomal peptide synthase domain TIGR01720/amino acid adenylation domain	6
**RAATTY (*Spirochaeta smaragdinae*)**	Upstream of transcriptional regulator, GntR family	4
**GATC (*Escherichia coli* CFT073)**	Upstream of Antigen 43 precursor	3*
	Upstream of TonB dependent receptor	3*
**GANTC (*Methylobacterium* sp.)**	Upstream of MobA/MobL family	3*
**TTAA (*Arthrobacter*)**	Upstream of Peptidase M60-like family / Protein-tyrosine-phosphatase	3
**TTAA (*Arthrobacter*)**	Upstream of Urease, gamma subunit / hypothetical protein	3

Umethylated *Dam* motif sites are located at predicted transcription factor binding sites (**S11 Fig**).

In summary, both known and novel orphan MTases are associated with a signature of unmethylated sites in regulatory regions of the genome. Many of these sites show evidence of evolutionary conservation and unmethylated sites are overall enriched near transcription start sites, both of which are hallmarks of gene regulatory sequences and support the notion that selective absence of methylation at MTase recognition sites plays a role in gene regulation.

### Identification of Orphan MTases with Putative Roles in Regulating DNA Replication

The orphan MTases Dam and CcrM are important regulators of genome replication in Proteobacteria. Regulation occurs through the differential recognition of fully methylated or hemi-methylated DNA by cellular machinery [35]. While such methylation patterns can in principle be determined from SMRT sequencing [21], it requires sampling of DNA from synchronized cells, which was not performed for our study. Nonetheless, the availability of large numbers of novel orphan MTase specificities makes it possible for us to search for general patterns of motif distribution (regardless of methylation state) consistent with a role in DNA replication control. We therefore systematically searched our methylome datasets for enriched clusters of motifs in non-coding regions of the genome. We restricted our analyses to conserved orphan MTases, and retained only those clusters of motifs that occur at orthologous locations in multiple organisms. As these analyses do not require methylome data, initial patterns of motif clusters were subject to expanded analyses of all publicly available genome sequences from related organisms (**Methods**).

**Fig 6:**
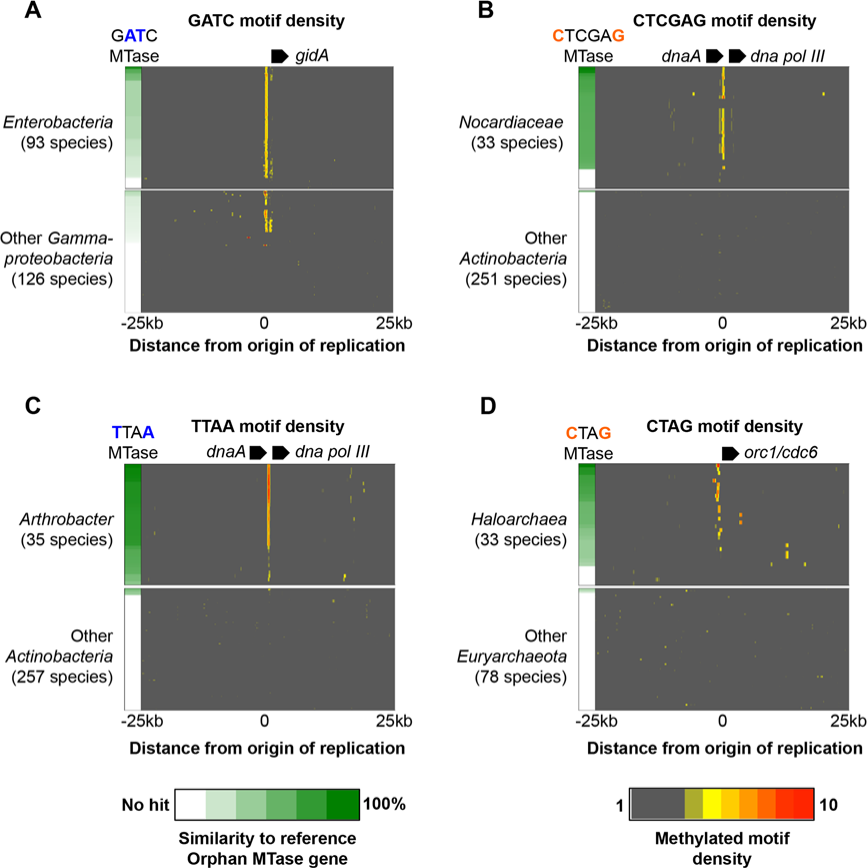
Identification of putative novel orphan MTase regulators of DNA replication. Four orphan MTases were found to be associated with enriched clusters of motifs in non-coding regions of the genome (**Methods**). Plots show density of motifs across a 50kb region of the genome flanking the motif cluster. Data is shown for the organism in which the pattern was originally identified, along with related organisms from the same taxonomic group. For comparison, plots were also generated from closely related organism lacking the orphan MTase. In each panel, a reference MTase sequence is selected (from **Fig 1**). The similarity score of the best scoring orthologs in each genome is represented in the MTase column using a white-green scale. **A**) Density of G**A**TC motifs flanking the *gidA* gene (origin of replication) in Enterobacteria and other Gammaproteobacteria. **B**) Density of CTCG**A**G motifs flanking the *dnaA* gene terminus in Nocardiaceae and other Actinobacteria species. **C**) Density of TTA**A** motifs flanking the dnaA gene terminus in Arthrobacter and other Actinobacteria species. **D**) Density of **C**TAG motifs flanking the *orc1/cdc6* gene start in Haloarchaea and other Euryarchaeota species (Bold and underlined characters indicate methylated bases. Underlined characters indicate reverse complement of methylated bases). In each example, the presence of motifs clusters correlates with the presence of the respective MTase in the same genome.

In total, we identified conserved clusters of motifs in non-coding regions of the genome in association with four orphan MTases (p < 1e-5, **Methods**). Strikingly, all cases were located at putative origins of replication (**Fig 6**). First, we observed enrichment of *Dam* MTase motifs at the origin of replication in Enterobacteria and other Gammaproteobacteria (**Fig 6A**). The presence of motif clusters correlates strongly with the presence of Dam orthologs in the genome, consistent with the known role of Dam in regulating DNA replication [17]. We observe similar patterns of motif enrichment for orphan MTases recognizing 5’-TTA^**m6**^**A**-3’ in *Arthrobacter*, and 5’-**C**TCGAG-3’ in *Nocardia* (Fig 6B and 6C respectively). In both cases, motif clusters occur in non-coding regions between bacterial replication genes *dnaA* and *dna polIII* [43]. Furthermore, the presence of motif clusters is again strongly correlated with the presence of the respective orphan MTase. Finally, we observe an analogous system associated with a conserved orphan MTase recognizing 5’-^**m4**^**C**ATG-3’ motifs in Haloarchaea (**Fig 6D**). In this case, motif clusters occur upstream of *orc6*/*cdc1* gene orthologs which encode the origin of replication complex in archaea [44]. Furthermore, motif clusters are frequently detected upstream of multiple *orc6*/*cdc1* genes in the same genome, consistent with the presence of multiple origins of replication [44]. Again, the presence of motif enrichment correlates with the presence of the orphan MTase. In summary, these analyses confirm a pattern of motif enrichment which co-occurs with the known regulators of DNA replication, and reveals three novel systems that share this pattern including an example of an orphan MTase with a potential role in regulating DNA replication or other functions in archaea.

## Discussion

Despite having potentially widespread functions, the global patterns of DNA methylation in prokaryotes are largely unexplored. Here, we obtain an initial overview of the epigenomic landscape of prokaryotes by single molecule sequencing the methylomes of 230 diverse bacteria and archaea. We find that methylation is pervasive, and present in at least 95% of the organisms we sequenced. We provide base-resolution methylation state information for every organism, and collectively identify over 800 methylated motifs, corresponding to the specificities of the MTases active in these organisms. Together these data massively expand the known repertoire of prokaryotic RM system specificities, and strongly suggest the presence of additional widespread functions of DNA methylation in prokaryotes.

SMRT sequencing offers a powerful approach to determine the recognition specificities of several Types of RM systems that have previously been very difficult to decipher. Type I RM systems cleave DNA at large distances from their binding site, while both Type IIG and Type III systems sometimes have difficulties in producing complete cleavage patterns. This can make them difficult to study using traditional approaches that rely on analysis of patterns of restriction digestion. However, in each of these RM system types, DNA methylation and restriction share specificity determinants such that identification of the MTase specificity automatically reveals the specificity of the cognate restriction enzyme. Furthermore, the type of methylation used by these RM systems is nearly always either m4C or m6A, both of which are readily detected by SMRT sequencing. Here, we realize this possibility by determining the specificities of 264 MTases from these RM systems, more than doubling the number of known specificities. Furthermore, the diversity of sequence specificities we reveal is astounding, with the vast majority (85%) of specificities currently unique.

Type II restriction enzymes have the property of recognizing a sequence and cleaving within or very close to it. This property provided a very simple experimental approach to specificity determination with the result that several thousand such specificities had been determined. Detailed experimental studies of a small number of examples suggested that their companion MTases would have the same specificity. In the present study, we show that this is generally true as abundantly exemplified by motifs recognized by Type II systems that have the clean specificity we have come to associate with Type II REases. Surprisingly, other than the Type IIG subtype, we detected very few new Type II MTase specificities, suggesting that extensive previous searches for Type II restriction enzymes for use as reagents already uncovered the majority of specificities that are present in nature. Since the phenomenon of increasing numbers of novel specificities being discovered is found among the Type I, Type IIG and Type III, but not Type II RM systems, the use of a single specificity system to guide both restriction and modification may be a key strategy employed by prokaryotes to build defense systems that ensure diversity.

Our analyses also reveal abundant DNA methylation occurring independently of RM systems. It is known that ‘orphan’ MTases are common in prokaryotic genomes, but beyond a handful of well-studied regulatory MTases there is little evidence that they are active, and the general importance of prokaryotic DNA methylation beyond RM systems remains unclear. Here, we confirm the activity and sequence specificity of over 100 novel orphan Type II MTases, with at least one such active gene detected in 48% of organisms, covering 15/20 (75%) phyla included in this study. Thus, there appears to be widespread prokaryotic DNA methylation beyond that involved in RM systems.

In general, orphan Type II MTases are associated with patterns of incomplete methylation of their target sites, clearly discriminating them from RM system MTases. The unmethylated sites associated with orphan MTases are frequently in non-coding sequences upstream of genes, thus hinting at potential regulatory roles. Furthermore, we frequently observe that both the orphan MTases and their associated patterns of methylation are conserved across related organisms. While the functions of these systems remain to be determined, we provide evidence that several MTases may function analogously to Dam and CcrM MTases and play a role in gene regulation. For example, we identify novel conserved MTases with putative gene regulatory roles in the phyla Spirochaetae, and Actinobacteria, and a conserved MTase family with a putative role in regulating DNA replication in Haloarchaea. To our knowledge, this is the first example of regulatory DNA methylation in Archaea. Together, this suggests that genome regulation may be one of the functions of the non-RM system DNA methylation that we observe.

There is reason to believe that the amount of DNA methylation, and its potential functions extend beyond those highlighted in our study. For example, Type II m5C MTases are abundant, and often appear to be orphans, but are not easily detectable by SMRT sequencing. Other MTases, such as those on prophages, appear to be inactive, but may be functional under other conditions. Furthermore, the functions of other Types of MTases may extend beyond their roles in restriction. For example, the functions of Type IIG systems are overall unclear but have recently been shown to include antiviral defense by a system that appears not to involve endonucleolytic cleavage [34]. Similarly, Type III restriction systems have been demonstrated to have important regulatory roles in phase variation [45–47]. The large numbers of novel RM systems and associated methylome data from this study will be a valuable resource for further exploration in this area.

Given the extensive amount of methylation present in the majority of the genomes we have examined, it is tempting to believe that methylation is a very important modification of bacterial and archaeal DNA perhaps providing regulatory functions that we have yet to fully appreciate. Additionally, it is reasonable to assume that the evolution of DNA methylation was an early event that was important for the viability of primitive organisms. Since methylation must have preceded the evolution of restriction enzymes, it is possible that restriction enzymes evolved not initially to provide protection against bacteriophages, but rather to ensure that the methylases remained active. Their value in protecting against external threats may have been a coincidental benefit. In this scenario DNA methylation suddenly becomes a key, yet still poorly understood, component of bacterial and archaeal life–one that perhaps plays a much deeper role in prokaryotic life than we currently appreciate.

In conclusion, methylome data is now easily obtained as a direct result of SMRT-sequencing, and potentially other technologies [48, 49]. Our study demonstrates the capacity of this approach to illuminate epigenomic phenomena in prokaryotes. Since many RM systems and orphan MTases are transferred from one organism to another by horizontal transfer [50], it seems likely that they will have significant effects on microbiomes. Our study highlights not only the importance of methylation studies, but provides initial insights into the kind of diversity that can be expected. There are undoubtedly some major discoveries to be made in this field as we delve into the details of methylation in individual organisms.

## Methods

### DNA Samples

The 230 target organisms were selected based on i) phylogenetic diversity, ii) relevance to D.O.E mission areas in bioenergy and the environment, iii) the presence of interesting and potentially interpretable RM systems. DNA samples were obtained from commercial sources (American Type Culture Collection, ATCC or DSMZ), or from contributions to the JGI community-sequencing program (http://jgi.doe.gov/collaborate-with-jgi/community-science-program/). A complete list of bacterial strain information and DNA sources is provided in **S1 Table**.

### Reference Genome Sequences and Annotation

All sequenced organisms have publicly available reference genome sequences, and gene annotation files deposited in NCBI and IMG [51]. Accession numbers and summary statistics of reference genomes used in the analyses are provided in **S1 Table**. The majority of reference sequences were complete (assembled into a single circular molecule), with more than 90% genomes containing less than 10 scaffolds. The inclusion of data from draft genome sequences increases our overall yield of MTase gene annotations, but limits the ability to comprehensively annotate the methylome in all cases.

### SMRT Sequencing

SMRT sequencing was performed using a library construction protocol described previously [52]. Libraries were sequenced on the Pacific Biosciences RS instrument using either C2, C3 or C4 chemistries. The average SMRT sequence coverage per genome was 130x (ranging from 31x to over 500x), with an average sub-read length of 1.8kb. Sequencing chemistries and sequencing yields for each DNA sample are summarized in **S1 Table**.

### Modification Detection and Motif Analysis

DNA modification detection and motif analysis were performed using the PacBio SMRT analysis platform (protocol version = 2.2.0 method = RS Modification and Motif Analysis.1, http://www.pacb.com/devnet/code.html). Briefly, raw reads were filtered using SFilter, to remove short reads and reads derived from sequencing adapters. Filtered reads were aligned to the reference genome using BLASR(v1) [53]. Modified sites were then identified through kinetic analysis of the aligned DNA sequence data [32]. Modified sites were then grouped into motifs using MotifFinder (v1)2. These motifs represent the recognition sequences of MTase genes active in the genome [54]. All kinetic data files have been deposited in GEO under accession numbers GSE69872, available for review using the following link: http://www.ncbi.nlm.nih.gov/geo/query/acc.cgi?token=ufapcaooxtcdbal&acc=GSE69872 The full list of identified modified motifs are in **S2 Table**.

### Annotation of Restriction Modification Genes

Restriction-Modification (RM) genes were assigned using the SEQWARE computer resource (Clark et al. 2012; Murray et al. 2012). It comprises a large suite of program modules with specialized databases containing compilations of protein sequences of bona-fide M system components as well as non-RM system components to weed out false positives. On a daily basis, newly published sequences are collected from GenBank and downloaded for analysis by SEQWARE and incorporation into the SEQWARE databases. Many routines that run in parallel scan the new data, queue different inspection steps, and depending on the preliminary findings, pipe data into further, more detailed analysis loops.

First, conserved elements of new RM systems are identified by sequence matches to known RM system genes. Most often these are MTases. Characteristic motifs [55, 56] of the newly detected items are located, and functional domains are mapped. From these, the Type and subtype of the inspected system are inferred, as well as the identity of potentially missing components–most often restriction enzyme genes. Genes for these missing components are then picked by a contextual analysis, where attribution is guided by marginal similarities in Type/subtype characteristic component order, while skipping genes that show better matches to non-RM system genes. Homologs that harbor non-RM functions (e.g. RNA MTases) generate many false hits in this first round of analysis, but are then filtered out by further matching to a library of known false positives. Occasionally, fusions of RM system genes to genes of unrelated enzymes are observed. To avoid false hits produced solely by the fused parts, the non-RM system domains are masked in the search library.

Newly detected systems are prepared for expert review. Items are annotated, and background supporting materials are prepared. These include hit lists, summary tables, schematics, plots and selectable alternatives for the resolution of undecided issues (e.g. handling of frame shifts). Following the curator’s decisions, results are recorded, and the internal databases are rebuilt for the next round of discovery. The program suite part of SEQWARE changes frequently as new modules are incorporated to accommodate new kinds of relevant data (shotgun, methylome), and as our understanding of RM systems expands. SEQWARE has been a prime supporting engine of restriction enzyme research for the last 20 years and is responsible for the bulk of the expansion of REBASE [5]. RM system gene annotations are summarized in **S2 Table**.

MTases without detectable RM genes in the flanking genome sequence were cautiously annotated as ‘candidate orphan’ MTases. Since restriction enzymes can be hard to identify, we cannot firmly conclude that a cognate restriction enzyme gene does not exist, and therefore some of these candidate orphan MTases may in reality be part of RM systems. However, our observation that many candidate orphan MTases exhibit incomplete modification of their genomes (**Fig 4**) is one line of evidence to suggest that the majority of these annotations are correct.

### Matching MTases to Methylated Motifs

In general, the sequence specificity of each putative MTase gene was predicted based on significant similarity to MTase genes of known specificity. Whenever such a gene was present and a motif of the same specificity was found, then the MTase gene was assumed to be responsible, unless more than one candidate MTase gene of the same specificity was present in which case no match was called. In the case of Type I, Type IIG and Type III genes in many cases only a single candidate gene was present for the particular kind of motif observed. Thus, for Type I genes, the recognition sequences are characteristically bipartite and usually asymmetric. For both Type IIG and Type III MTases methylation is only present on one strand. Again if only one gene was present then it could be matched unambiguously to the motif. In some cases, for Type I systems one half of the recognition sequence would match half of a known specificity in another organism. Often, this would then permit matching of the appropriate S subunit to the motif. In all cases where there were no clear and unambiguous matches, the motif was marked as unmatched (see **S3 Table** and **S4 Table**). In some cases reasonable guesses could be made and these are indicated in **S3 Table** and **S4 Table** by putting the motifs and the genes likely to match them in parentheses.

### Experimental Characterization of Individual RM System Components

Nine enzymes have been characterized as restriction enzymes. Three typical Type IIG single polypeptide REase-MTase proteins have been cloned and their recognition motif determined from their endonuclease cleavage patterns: SdeAI and PliMI from the MmeI family, and RpaI, a representative of the TaqII family [33]. Similarly the endonuclease genes for the MjaI, MjaII, MjaIII, MjaIV and MjaV systems from *Methanocaldococcus jannachii* DSM 2661 as well as Csp12AI from *Clostridium sp*. 12(A), have been cloned, expressed and characterized through their endonuclease activity. The modified base and recognition motif for three MTases by cloning the MTase gene into the non-modifying host ER2796 followed by SMRT sequencing and analysis using methods described in Murray et al. 2012 (8). In this way we also characterized the Type III MTase M.Nme18I which recognizes AC^m6^ACC [27], and M1.Csp12AI and M2.Csp12AI from *Clostridium sp*. 12(A).

### Conservation Analysis of Type II MTases

Amino acid sequences of all annotated Type II MTases were obtained from IMG and used as queries in BLASTP (blastall v2.2.26) searches of protein sequence databases of 35,184 bacterial and archaeal genomes in IMG. Database hits with a similarity score of 35 or more (where similarity score = 100*(bitscore of hit to database / bitscore of hit to self)) were considered potential orthologs of the MTase. Taxonomy information for all database genomes was also obtained from IMG, and used to determine the fraction of organisms across each taxonomic category with a potential MTase ortholog. MTases with orthologs in >50% of species from a taxonomic group were considered ‘conserved’.

### Identification of Type II Orphan MTase Families

Amino acid sequences of all annotated Type II MTases were obtained from IMG, and split into two groups according to base methylation type (m6A or m4C and m5C). For each group, all versus all alignments were performed with usearch, v8.0.1616_i86linux32 [57], using the search_global command (with parameters–fulldp–id 0 –uc). Initial clusters of related MTases were identified using usearch–cluster_agg (with parameters -id 0.35 -linkage min–fulldp. Using custom perl scripts, MTases were annotated with taxonomic classification of the host organism, presence or absence of cognate REase, and motif specificity. Annotated MTase clusters were then manually inspected to identify individual sub-clusters of orphan MTases with identical or closely related specificities from taxonomically related organisms. The resulting sub-clusters represent putative orphan MTase families.

### Identification of Unmethylated Motifs

The ipdR (inter-pulse duration ratio) is the primary metric in DNA modification detection. It corresponds to the time delay in incorporation of successive bases in a sample versus an unmodified control. Unmethylated motifs were identified using inter pulse duration ratio (ipdR) measurements [32], and read coverage. For each methylated motif, an ‘under-methylated’ ipdR threshold was determined by comparison of ipdR scores of bases in methylated motifs with those in unmethylated, non-motif sequences. ipdR scores for all motif sites in the genome were ranked, and an average motif ipdR calculated across the central 60% values (to minimize the effect of unmethylated sites or other outliers). The average non-motif ipdR was similarly calculated from the central 60% of ranked ipdR scores from all bases of the same type in non-motif sequences in the genome. The under-methylated ipdR threshold was then defined as (0.1*average motif ipdR)+(0.9*average non-motif ipdR), i.e. an approximation of the idpR score if 10% of bases were methylated. For comparison, a ‘methylated’ ipdR threshold was defined as (0.5*average motif ipdR)+(0.5*average non-motif ipdR), i.e. an approximation of the idpR score if at least half of bases were methylated. Analysis of unmethylated motifs was only performed for palindromic Type II motifs that have two methylated sites (one on each strand of the genome). Motif instances were considered ‘unmethylated’ if both potential methylated bases had at least twenty-fold SMRT sequence coverage, and an ipdR less than the ‘under-methylated’ threshold. Importantly, the average SMRT sequence coverage at unmethylated sites is no different from that at methylated sites (**S4 Fig**). They are therefore high-confidence unmethylated sites, and not simply borderline cases at the thresholds for inclusion in the analysis.

### Analysis of Unmethylated Motif Enrichment Near Upstream Gene Regulatory Regions

Gene regulatory regions were defined as 100bp upstream of the CDS start to 50bp downstream of the CDS start. Fold enrichment of all motif sequences in gene regulatory regions was determined by comparison with the average fraction of randomized control sequences in regulatory regions (1000 random samplings of an equal number of sites in the genome with the same length and nucleotide composition as the modified motif). Fold enrichment of unmethylated motifs in gene regulatory regions was determined by comparison with the fraction of methylated motifs in regulatory regions (sites with ipdR scores greater than the ‘methylated’ threshold defined above). Significance of enrichment was determined using Fisher’s exact test. To determine the potential enrichment of unmethylated motifs for specific functional classes of genes, we repeated these analyses using individual subsets of regulatory regions grouped according to the COG category annotations [58] of their corresponding genes.

### Identification of Conserved Clustered Unmethylated Sites

For each orphan MTase family associated with incomplete methylation of the genome (**Fig 4**), we identified conserved unmethylated sites based on conservation of flanking gene sequences (**Table 1**). For every unmethylated site, we took the amino acid sequences of the two flanking genes, and identified best hits in each of the other genomes using BLASTP. Pairs of unmethylated sites across the two genomes were considered conserved if their flanking genes were reciprocal best hits in the respective other genome. For select conserved sites, genomic DNA sequences upstream of the putative target gene were subject to multiple alignment using MAFFT [59] and visualized in Jalview [60].

### Overlap between Unmethylated Motifs and Transcription Factor Binding Sites

To identify potential overlap between unmethylated *Dam* GATC motifs and transcription factor binding sites, we obtained curated transcription factor binding site (TFBS) probability matrices from http://regtransbase.lbl.gov [61], and searched for matches to these matrices using MAST [62]. We restricted our search to TFBS that were identified in gammaproteobaceria (using the taxonomic classifications provided by regtransbase), and to gammaproteobacerial genomes from our study that contain at least 4 unmethylated Dam motif sites. We identified overlap between predicted TFBSs and unmethylated Dam motifs using bedtools [63].

### Identification of Clustered Unmethylated Sites

Candidate enriched clusters of unmethylated motifs were identified as regions of the genome containing at least 3 consecutive unmethylated motifs, each separated from its nearest neighbor by less than the genome-wide average distance between motifs (**Table 2**). A cluster of unmethylated sites was considered significant if the probability of observing such a series of consecutive sites by chance was < = 0.01, based on 10,000 iterations of randomly sampling *n* = (number of unmethylated sites) times from an ordered list of length *l* = (total motif sites).

### Identification of Motif Clusters in Non-coding Regions of the Genome

We searched for unusual clusters of motifs (regardless of methylation state) in non-coding regions of the genome (defined using IMG gene annotations of coding DNA sequences, and excluding RNA gene annotations). For each non-coding region, we calculated the local non-coding motif density (motifs / bp) across the 100 flanking non-coding regions. The expected number of motifs in that region was then estimated as (non-coding region length)*(local motif density). Fold enrichment was determined as observed number of motifs / expected number of motifs. P-values were determined using the pbinom function in R (p_val = 1-pbinom (observed number of motifs, non-coding region length, expected number of motifs)), and subject to Bonferroni correction using the number of non-coding regions in the genome. A p-value threshold of 1e-5 was used to identify non-coding regions with motif enrichment. We identified patterns of non-coding motif enrichment conserved across organisms, based on reciprocal best BLAST hits of flanking genes (as described above).

For methylated motifs showing conserved patterns of motif enrichment, we extended our analyses to other sequenced genomes in the same taxonomic class (**Fig 6**). For each genome, we calculated motif density in 500bp windows with a 50bp step-size across a 50kb region of the genome centered on the start or end of the gene closest to the motif cluster. BLAST was used to search each genome for orthologs of the responsible MTase, and determine correlation between presence of MTase and non-coding motif clusters.

## Supporting Information

S1 Fig Breakdown of base methylation type for **(A)** Observed specificities (by SMRT Sequencing) of all functionally annotated MTases. **(B)** Predicted specificities (based on homology to other MTases in REBASE) of all candidate MTases without detectable activity.
(TIF)

S2 Fig Distribution of mean ipdR scores for each modified base type. The ipdR (inter-pulse duration ratio) is the primary metric in DNA modification detection. It corresponds to the time delay in incorporation of successive bases in a sample versus an unmodified control. For each methylated motif, the mean ipdR across all motif instances is calculated as a summary statistic. Here we show the distribution of mean ipdR across all motifs in our dataset by base modification type. Boxes represent the median and 25 and 75 percentiles, ‘whiskers’ represent the 5 and 95 percentiles. The typical mean ipdR ratios for m6A and m4C are greater than for m5C, reflecting the known differences in sensitivity to these modification types on the Pacific Biosciences sequencing platform. For comparison, we also show the distribution of mean ipdR at unmodified sites in each genome.
(TIF)

S3 Fig (**A**) Breakdown of all identified MTases by RM system type. (**B**) Breakdown of all detected methylated motifs by RM system type. Illustrative examples are shown for each methylated motif type are shown. Names are of the matched MTase gene in REBASE.
(TIF)

S4 Fig Examples of novel Type IIG-like RM systems discovered in this study. We identified several novel RM systems with overall similarity to Type IIG systems but with the MTases and REases atypically encoded on separate peptides. **A.** BloAII is representative of a typical MmeI-like Type IIG system [33] consisting of a single polypeptide with an N-terminal PD-ExK endonuclease domain, an m6A MTase, and a C-terminal DNA recognition domain. **B.** AchA6III has 9 RM orthologs in REBASE. It has one gene that matches the MTase/TRD in the same overall architecture as MmeI, but which lacks the PD-ExK endonuclease motif. Instead, an adjacent gene contains a fused PLD-family endonuclease/translocase. **C.** OspHL35III is shown as the prototypical representative of several hundred members in REBASE. It has three separate proteins, consisting of a MTase having methylase/specificity domains like MmeI but lacking the N-terminal endonuclease motif together with separate translocase and GIY-YIG family endonuclease proteins. **D**. CalB3II appears to be similar to BREX-like systems [34]. These multi-protein BREX systems use the specific methylation of the MTase protein to distinguish self from non-self in phage restriction, but appear to accomplish restriction without generating DNA cleavage. **E.** MexAMORF1192P is a representative RM system that is unrelated to MmeI or BREX. It is characterized by two translocase proteins flanking the MTase-TRD protein, one of which has a putative metal binding domain (DUF1998) at its C-terminus, together with a separate HNH family endonuclease protein. Work to characterize and formally classify the Type IIG like systems shown here is ongoing, and will be provided in updates to the REBASE database [5].
(TIF)

S5 Fig SMRT analysis of methylated motifs present when MTases M1.Csp12AI and M2.Csp12AI (from *Clostridium sp*. 12A) were cloned in the DNA methyltransferase deficient *E*. *coli* strain ER 2796. A. Methylated base in the motif C^m6^ATCC detected when M2.Csp12AI was cloned alone. B. Methylated bases detected when M1.Csp12AI and M2.Csp12AI were cloned together. The specificity of M1.Csp12AI was deduced to be GG^m6^ATG. (M1.Csp12AI could not be cloned alone as it proved toxic to E. coli).
(TIF)

S6 Fig (**A**) Schematic diagram of the 1.2 Mb segment of the *Clostridium sp*. *12A* separating the M and R genes of the Csp12AI system. (**B**) Results from PCR amplification of M1.Csp12AI, M2.Csp12AI and Csp12AI. (Mr = Molecular weight marker. M1 = PCR amplification of the M1 gene using flanking primers as indicated. M2 = PCR amplification of the M2 gene using flanking primers as indicated. M12 = PCR amplification of the M1 and M2 genes using flanking primers as indicated. Lc = PCR amplification from a primer located with the M2 gene and a second primer located 16kb downstream. The band marked with the arrow is an artefact. M2R = PCR amplification from a primer located with the M2 gene and a second primer located within the R gene (1.2 Mb apart based on the published sequence). There is no product because the distance is too long for robust PCR. The band marked with the arrow is an artefact also present in Lc. Rc = PCR amplification from a primer located within the R gene and a second primer located 15.7kb upstream.
(TIF)

S7 Fig Identification of families of orthologous orphan Type II MTases (m6A). Amino acid sequence similarity was calculated for all pairs of m6A MTase with an annotated motif, and the resulting matrix of similarity scores clustered heirarchically. Each row / column corresponds to an MTase, and rows and columns are identical ordered. The matrix indicates pairwise amino acid sequence similarity from 35% (yellow) to 100% (red). Each MTase row is labeled with motif specificity, the class of organism in which the MTase is found and whether the MTase has a cognate REase (red) or is an ‘orphan’ (blue). This approach results in accurate grouping of MTases by sequence specificity. For example, the prominent cluster at the top of the figure corresponds to Ccrm orthologs, while that at the bottom corresponds to *Dam* methylases. To identify candidate orphan MTase families, we manually identified sub-clusters of orphan MTases with identical or closely related specificities from taxonomically related organisms. Candidate families are indicated by blue outer lines in the matrix plot.
(TIF)

S8 Fig Identification of families of orthologous orphan Type II MTases (m4C and m5C). Amino acid sequence similarity was calculated for all pairs of m4C and m5C MTase with an annotated motif, and the resulting matrix of similarity scores clustered heirarchically. Each row / column corresponds to an MTase, and rows and columns are identical ordered. The matrix indicates pairwise amino acid sequence similarity from 35% (yellow) to 100% (red). Each MTase row is labeled with motif specificity, the class of organism in which the MTase is found and whether the MTase has a cognate REase (red) or is an ‘orphan’ (blue). This approach results in accurate grouping of MTases by sequence specificity. To identify candidate orphan MTase families, we manually identified sub-clusters of orphan MTases with identical or closely related specificities from taxonomically related organisms. Candidate families are indicated by blue outer lines in the matrix plot.
(TIF)

S9 Fig Comparison of SMRT sequence coverage at methylated and unmethylated motif sites. Plots indicate median, 25–75 percentile (boxes) and 5–95 percentile (whiskers) SMRT sequence coverage at methylated and unmethylated motifs, targeted by four different orphan MTases. In all cases, coverage at unmethylated sites is equivalent to or slightly greater than that at methylated sites, suggesting that the unmethylated sites associated with orphan MTases are not simply a consequence of low sequence coverage.
(TIF)

S10 Fig Overlap between unmethylated *dam* methylase motifs and predicted transcription factor binding sites in Gammaproteobacteria. For each gammaproteobacteria with at least 5 unmethylated *dam* motif sites, we calculated overlap between GATC motifs and predicted transcription factor binding sites (TFBSs) in the same genome. Black bars represent proportion of unmethylated GATC motifs overlapping a TFBS. For comparison, gray bars indicate proportion of methylated GATC motifs overlapping a TFBS. The number of overlapping and non-overlapping GATC motifs is shown above each bar. In many organisms, unmethylated motifs overlap a TFBS significantly more frequently than do methylated motifs (*, p<0.05, fishers exact test).
(TIF)

S11 Fig Conserved unmethylated sites at the PTS locus in Gammaproteobacteria. Upper panel shows a multiple sequence alignment of 100 Enterobacteria species to a 100bp sequence upstream of the PTS system in *E*. *coli*. Blue asterisk indicates the location of a conserved undermethylated site observed in 6 enterobacteria species in this study. Bar charts show the extent of DNA methylation (inter-pulse duration ratio) at the candidate regulatory unmethylated site (blue) and, for comparison, at the ten immediately flanking upstream and downstream GATC motifs (red).
(TIF)

S1 Table Table of all 230 organisms, summary of SMRT sequencing and motif counts. (XLSX)

S2 Table Organisms with no detected DNA methylation. Organisms without detected modification were from across the sampled taxa. In many cases the absence of methylation correlates with the absence of MTase genes in the genome. In other cases, MTases are present but not detected by SMRTsequencing. Sequencing coverage was on average higher for this set of samples, than for the study as a whole.
(DOCX)

S3 Table Table of all detected modified motifs. (XLSX)

S4 Table Table of all detected MTases. (XLSX)

S5 Table Table of conserved unmethylated motifs. (XLSX)

S1 Text Descriptions of Type I and Type III orphan MTases. (DOCX)
