# Correction: The Epigenomic Landscape of Prokaryotes

**DOI:** 10.1371/journal.pgen.1006064

**Published:** 2016-05-12

**Authors:** The PLOS Genetics Staff

There is an error in affiliation for Editor Gang Fang. The affiliation should be:

Icahn School of Medicine at Mount Sinai, UNITED STATES
