# Supplementary figures and images for: The Epigenomic Landscape of Prokaryotes
Source: PLoS Genet. 2016 Feb 12;12(2):e1005854. doi: 10.1371/journal.pgen.1005854 (PMC4752239; doi:10.1371/journal.pgen.1005854)

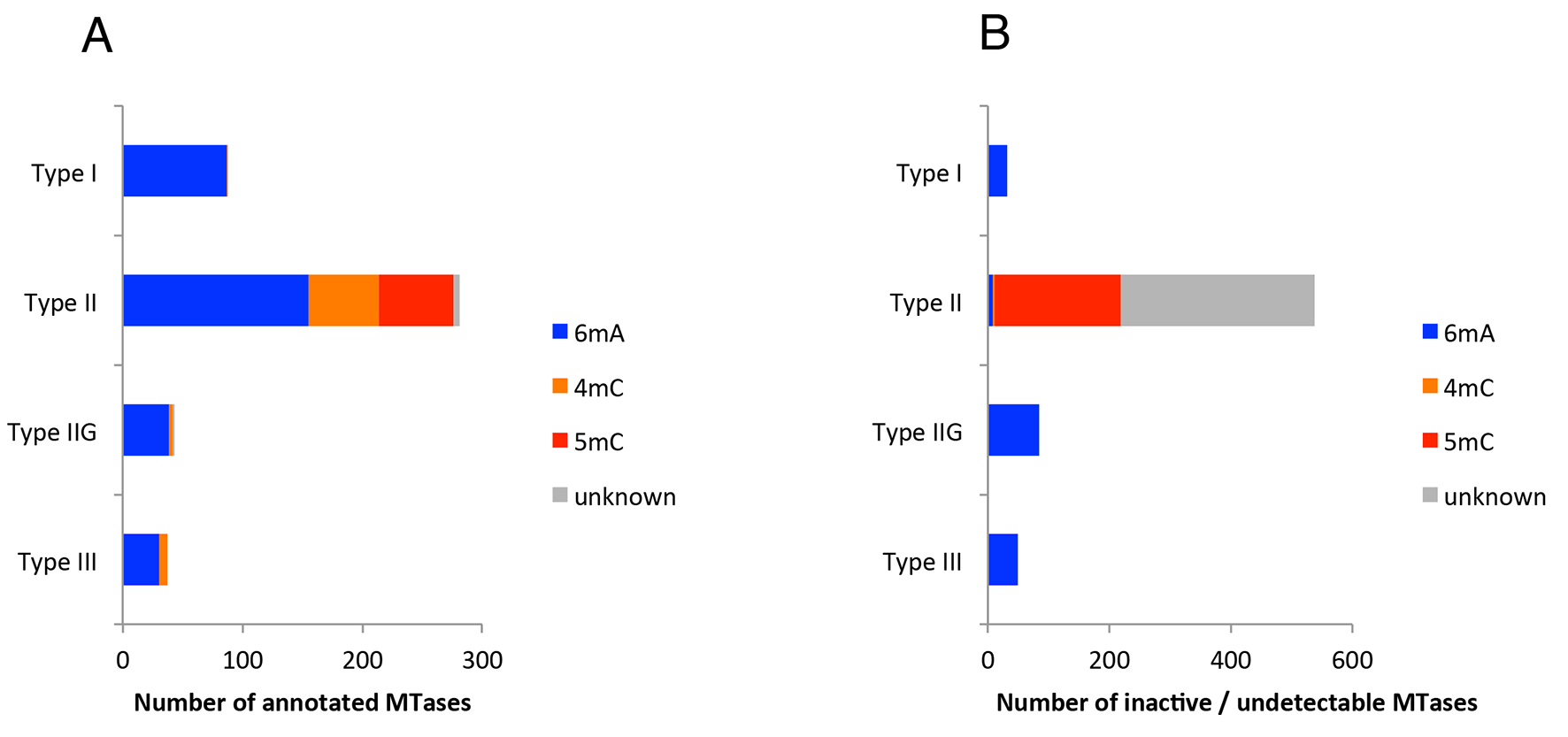

Supplement: S1 Fig — Breakdown of base methylation type for (A) Observed specificities (by SMRT Sequencing) of all functionally annotated MTases. (B) Predicted specificities (based on homology to other MTases in REBASE) of all candidate MTases without detectable activity. (TIF) [file pgen.1005854.s001.tif]

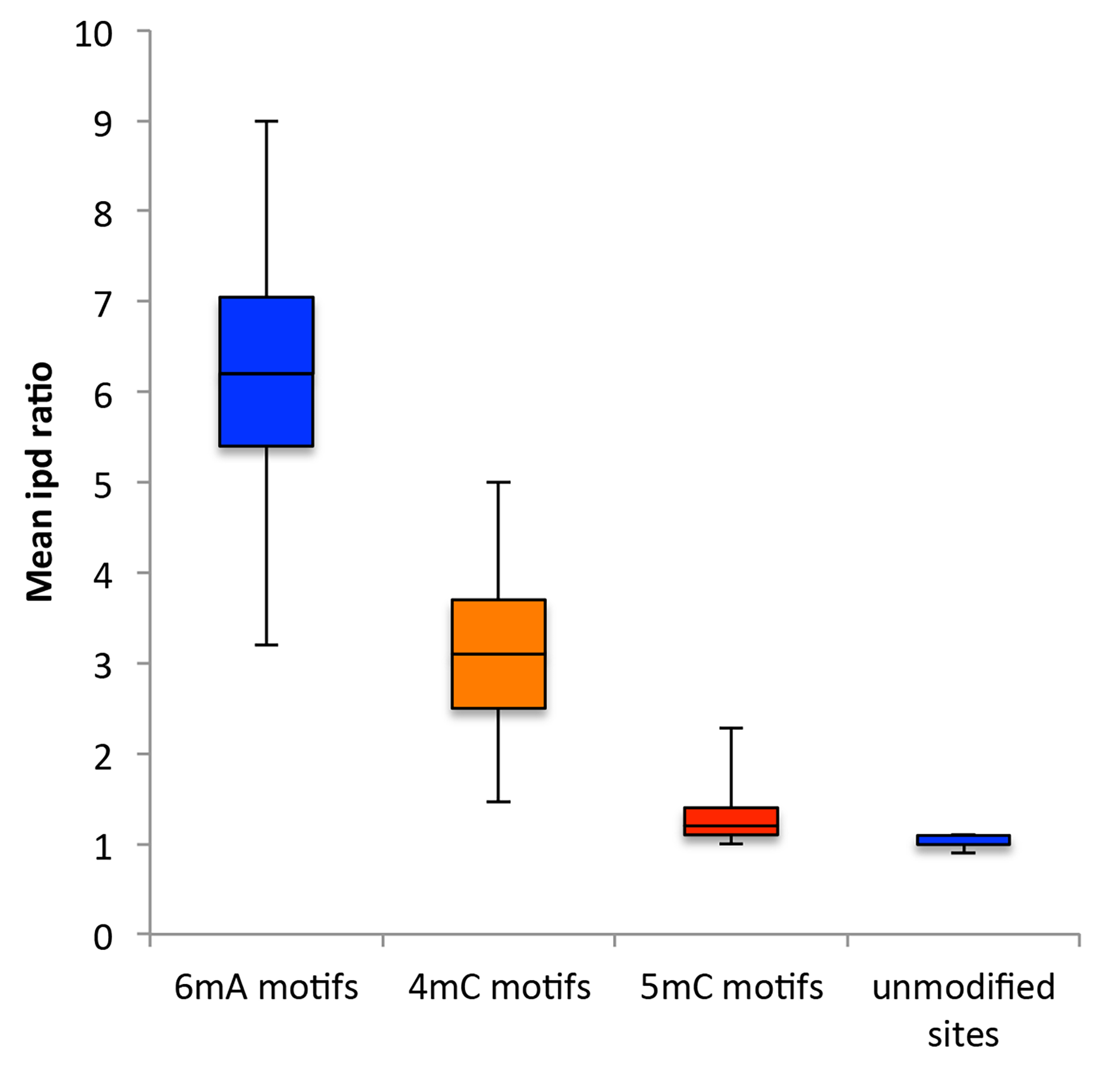

Supplement: S2 Fig — The ipdR (inter-pulse duration ratio) is the primary metric in DNA modification detection. It corresponds to the time delay in incorporation of successive bases in a sample versus an unmodified control. For each methylated motif, the mean ipdR across all motif instances is calculated as a summary statistic. Here we show the distribution of mean ipdR across all motifs in our dataset by base modification type. Boxes represent the median and 25 and 75 percentiles, ‘whiskers’ represent the 5 and 95 percentiles. The typical mean ipdR ratios for m6A and m4C are greater than for m5C, reflecting the known differences in sensitivity to these modification types on the Pacific Biosciences sequencing platform. For comparison, we also show the distribution of mean ipdR at unmodified sites in each genome. (TIF) [file pgen.1005854.s002.tif]

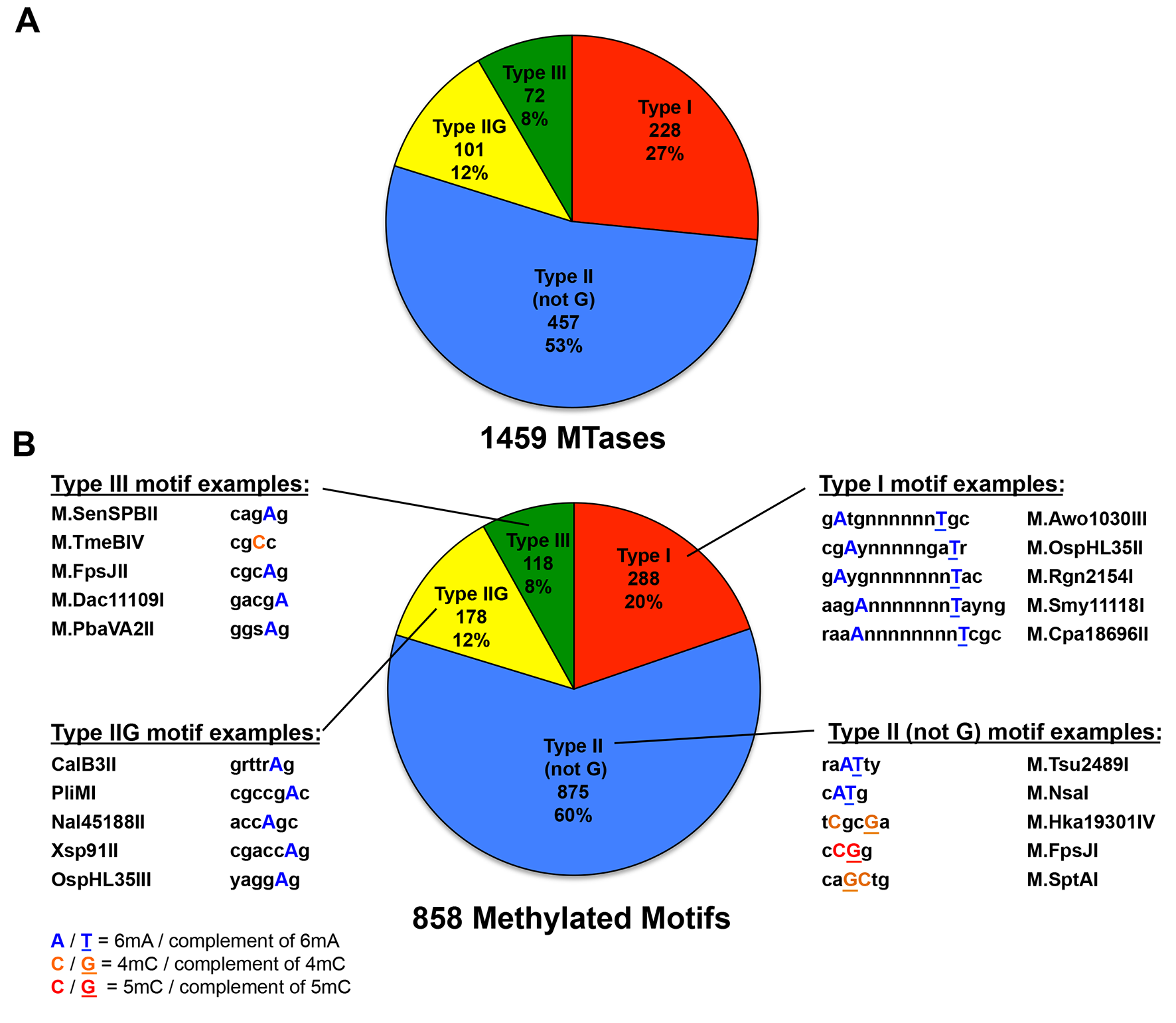

Supplement: S3 Fig — (A) Breakdown of all identified MTases by RM system type. (B) Breakdown of all detected methylated motifs by RM system type. Illustrative examples are shown for each methylated motif type are shown. Names are of the matched MTase gene in REBASE. (TIF) [file pgen.1005854.s003.tif]

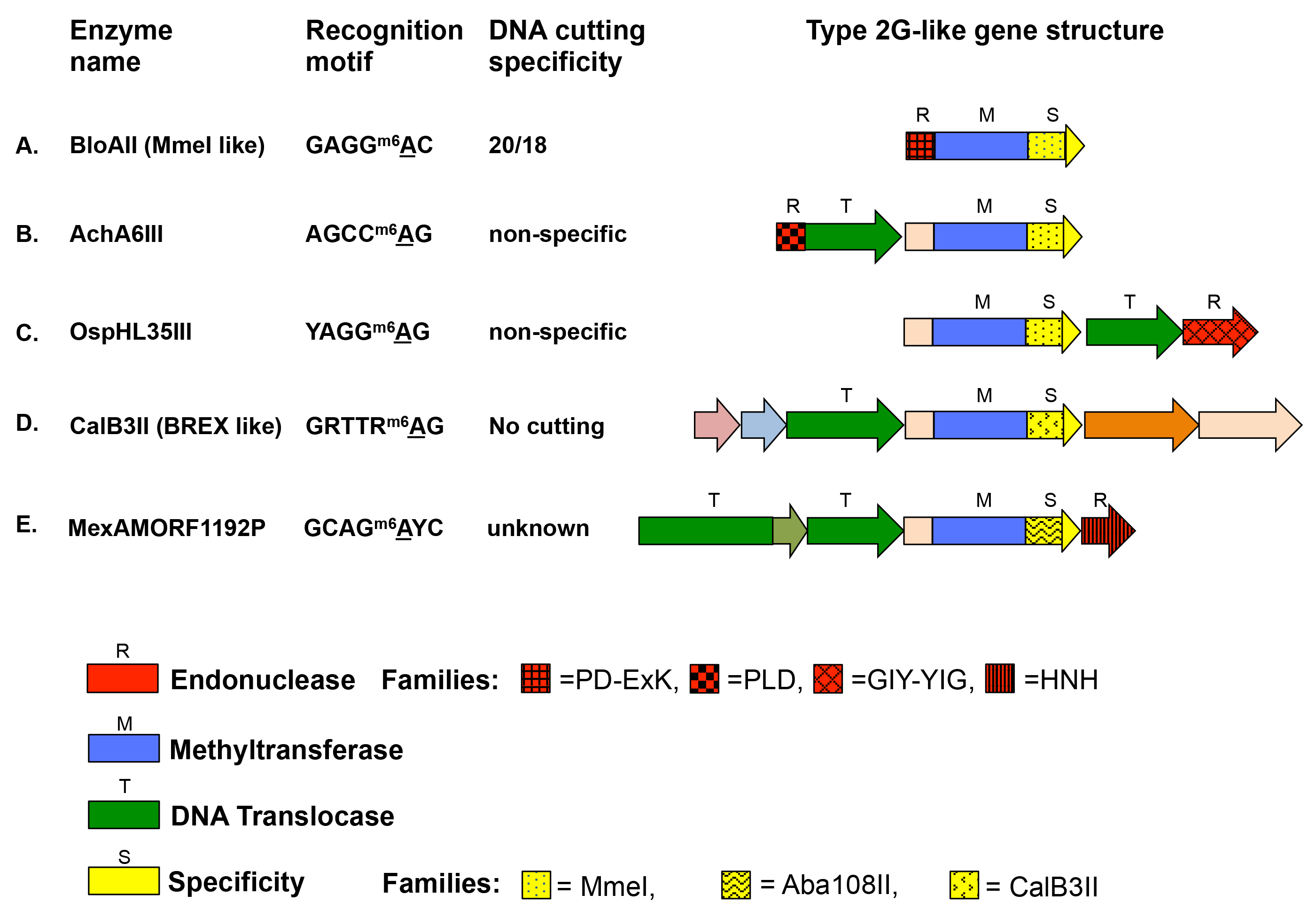

Supplement: S4 Fig — We identified several novel RM systems with overall similarity to Type IIG systems but with the MTases and REases atypically encoded on separate peptides. A. BloAII is representative of a typical MmeI-like Type IIG system [33] consisting of a single polypeptide with an N-terminal PD-ExK endonuclease domain, an m6A MTase, and a C-terminal DNA recognition domain. B. AchA6III has 9 RM orthologs in REBASE. It has one gene that matches the MTase/TRD in the same overall architecture as MmeI, but which lacks the PD-ExK endonuclease motif. Instead, an adjacent gene contains a fused PLD-family endonuclease/translocase. C. OspHL35III is shown as the prototypical representative of several hundred members in REBASE. It has three separate proteins, consisting of a MTase having methylase/specificity domains like MmeI but lacking the N-terminal endonuclease motif together with separate translocase and GIY-YIG family endonuclease proteins. D. CalB3II appears to be similar to BREX-like systems [34]. These multi-protein BREX systems use the specific methylation of the MTase protein to distinguish self from non-self in phage restriction, but appear to accomplish restriction without generating DNA cleavage. E. MexAMORF1192P is a representative RM system that is unrelated to MmeI or BREX. It is characterized by two translocase proteins flanking the MTase-TRD protein, one of which has a putative metal binding domain (DUF1998) at its C-terminus, together with a separate HNH family endonuclease protein. Work to characterize and formally classify the Type IIG like systems shown here is ongoing, and will be provided in updates to the REBASE database [5]. (TIF) [file pgen.1005854.s004.tif]

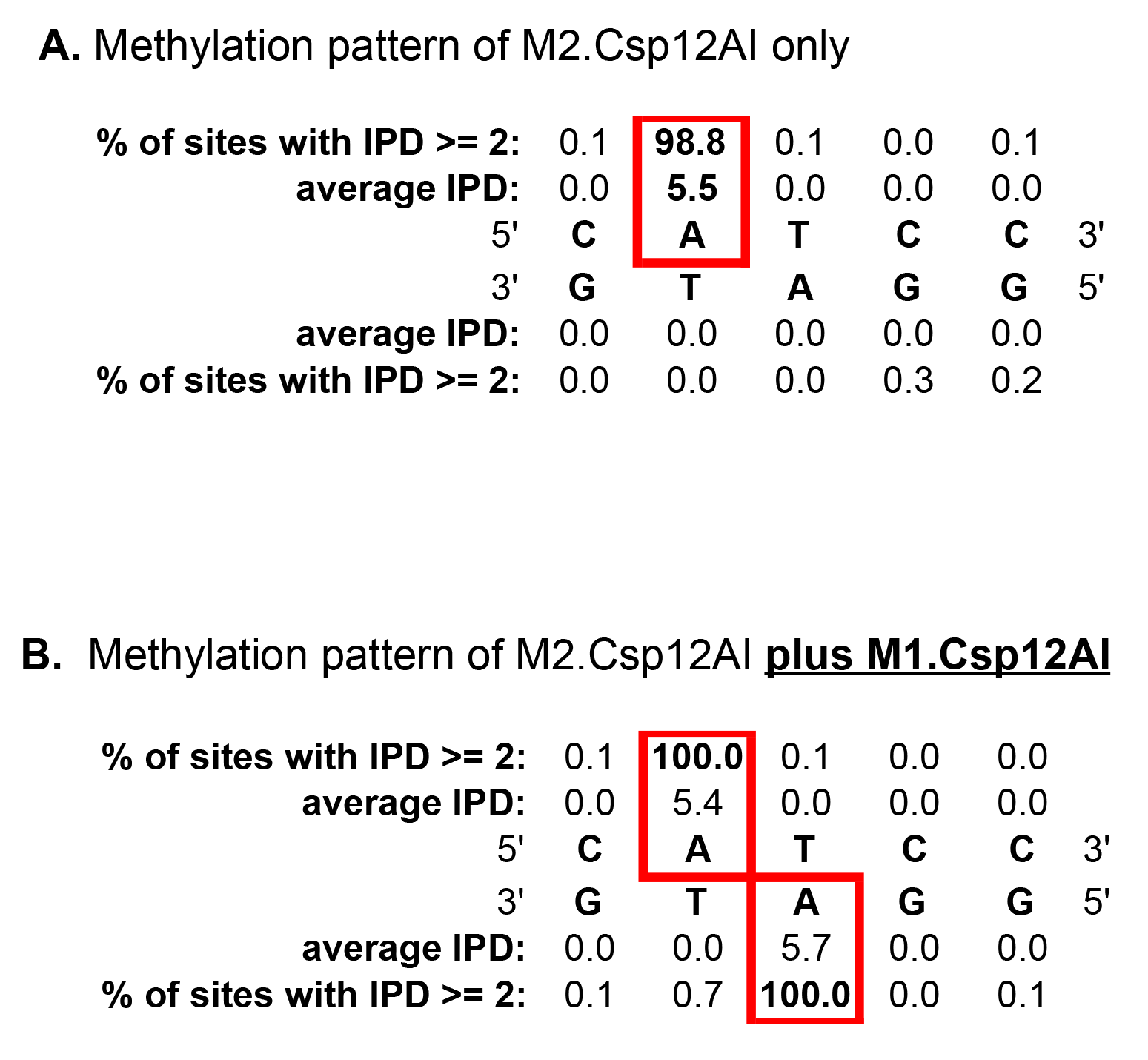

Supplement: S5 Fig — A. Methylated base in the motif Cm6ATCC detected when M2.Csp12AI was cloned alone. B. Methylated bases detected when M1.Csp12AI and M2.Csp12AI were cloned together. The specificity of M1.Csp12AI was deduced to be GGm6ATG. (M1.Csp12AI could not be cloned alone as it proved toxic to E. coli). (TIF) [file pgen.1005854.s005.tif]

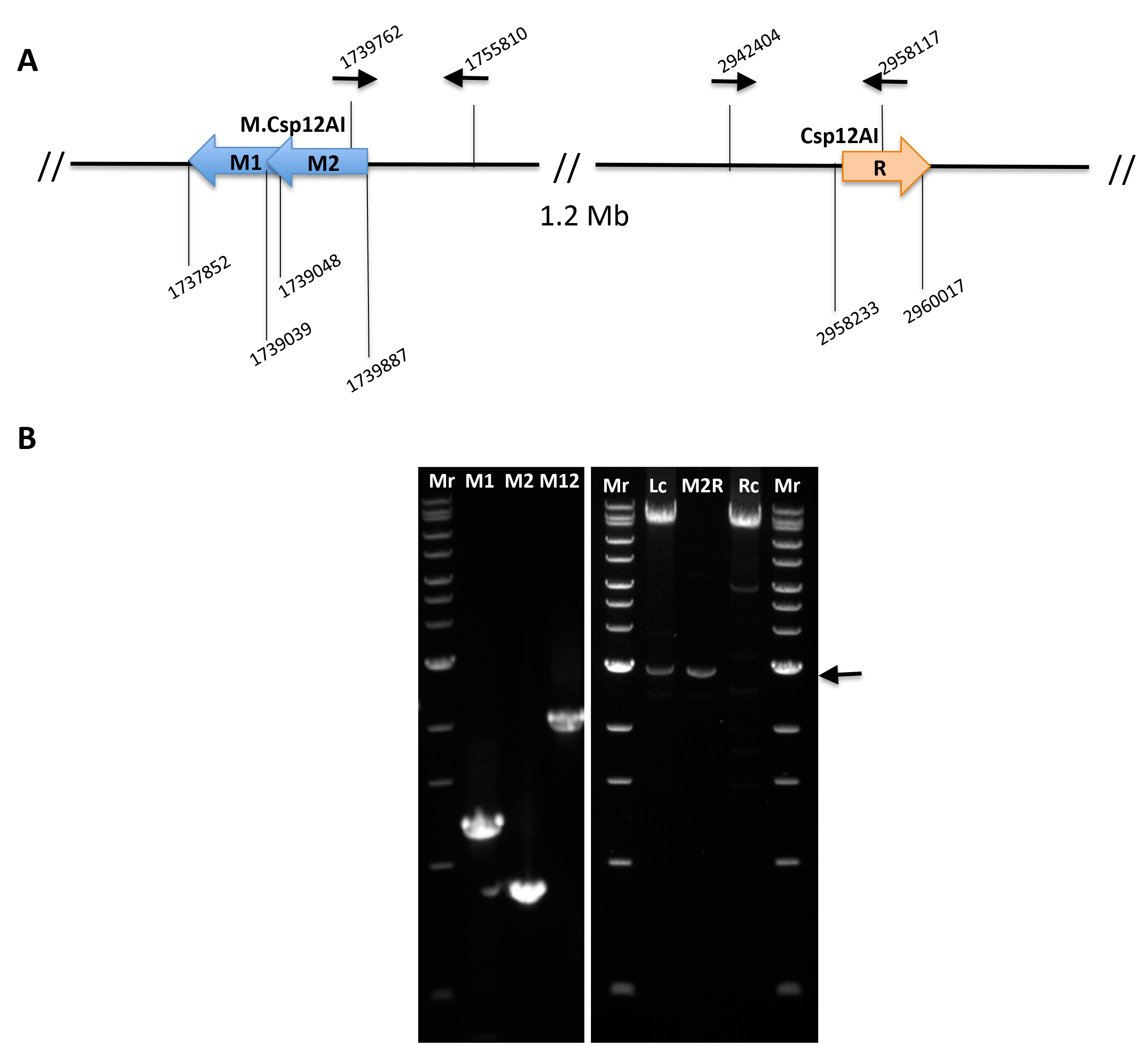

Supplement: S6 Fig — (A) Schematic diagram of the 1.2 Mb segment of the Clostridium sp. 12A separating the M and R genes of the Csp12AI system. (B) Results from PCR amplification of M1.Csp12AI, M2.Csp12AI and Csp12AI. (Mr = Molecular weight marker. M1 = PCR amplification of the M1 gene using flanking primers as indicated. M2 = PCR amplification of the M2 gene using flanking primers as indicated. M12 = PCR amplification of the M1 and M2 genes using flanking primers as indicated. Lc = PCR amplification from a primer located with the M2 gene and a second primer located 16kb downstream. The band marked with the arrow is an artefact. M2R = PCR amplification from a primer located with the M2 gene and a second primer located within the R gene (1.2 Mb apart based on the published sequence). There is no product because the distance is too long for robust PCR. The band marked with the arrow is an artefact also present in Lc. Rc = PCR amplification from a primer located within the R gene and a second primer located 15.7kb upstream. (TIF) [file pgen.1005854.s006.tif]

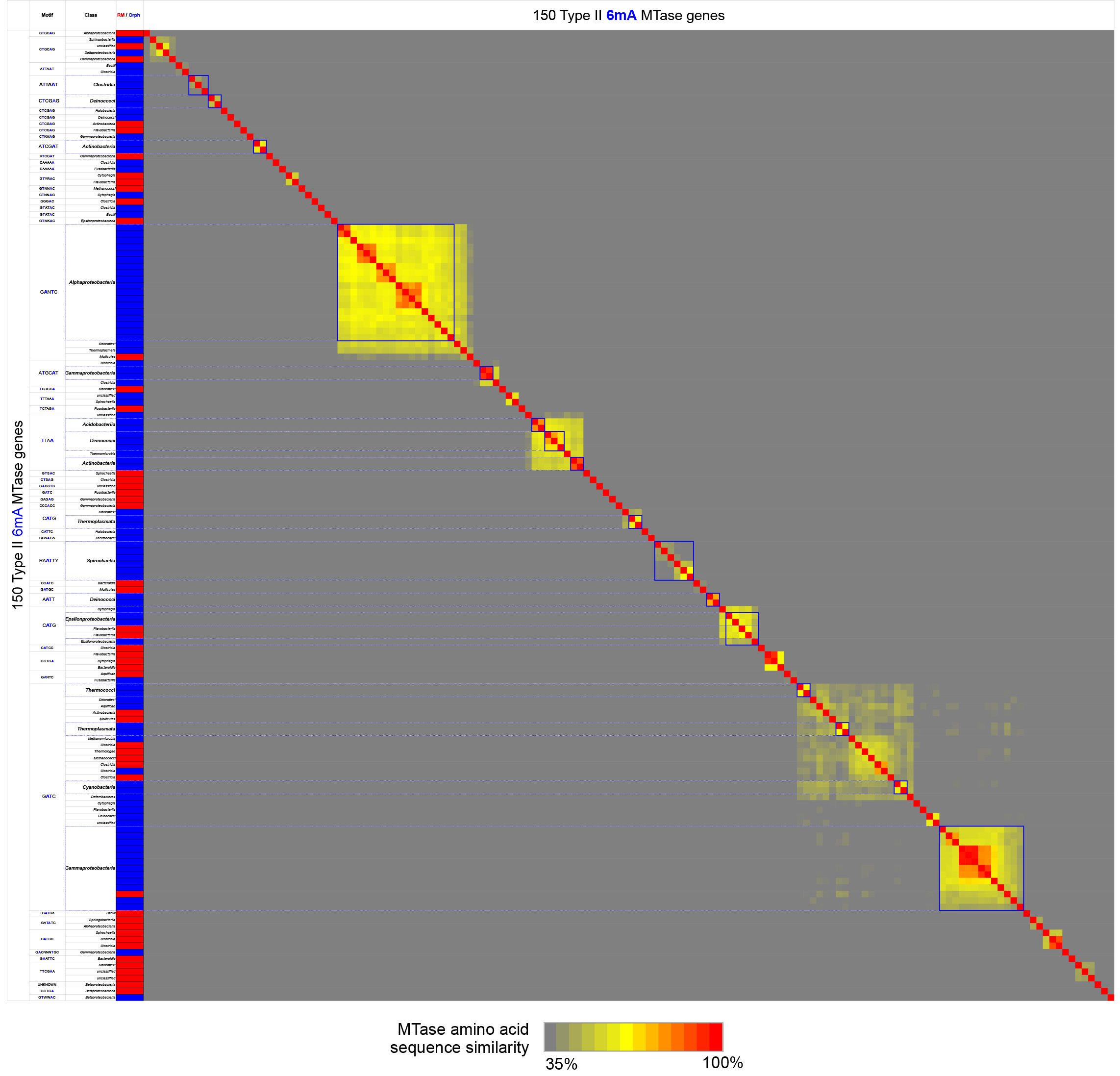

Supplement: S7 Fig — Amino acid sequence similarity was calculated for all pairs of m6A MTase with an annotated motif, and the resulting matrix of similarity scores clustered heirarchically. Each row / column corresponds to an MTase, and rows and columns are identical ordered. The matrix indicates pairwise amino acid sequence similarity from 35% (yellow) to 100% (red). Each MTase row is labeled with motif specificity, the class of organism in which the MTase is found and whether the MTase has a cognate REase (red) or is an ‘orphan’ (blue). This approach results in accurate grouping of MTases by sequence specificity. For example, the prominent cluster at the top of the figure corresponds to Ccrm orthologs, while that at the bottom corresponds to Dam methylases. To identify candidate orphan MTase families, we manually identified sub-clusters of orphan MTases with identical or closely related specificities from taxonomically related organisms. Candidate families are indicated by blue outer lines in the matrix plot. (TIF) [file pgen.1005854.s007.tif]

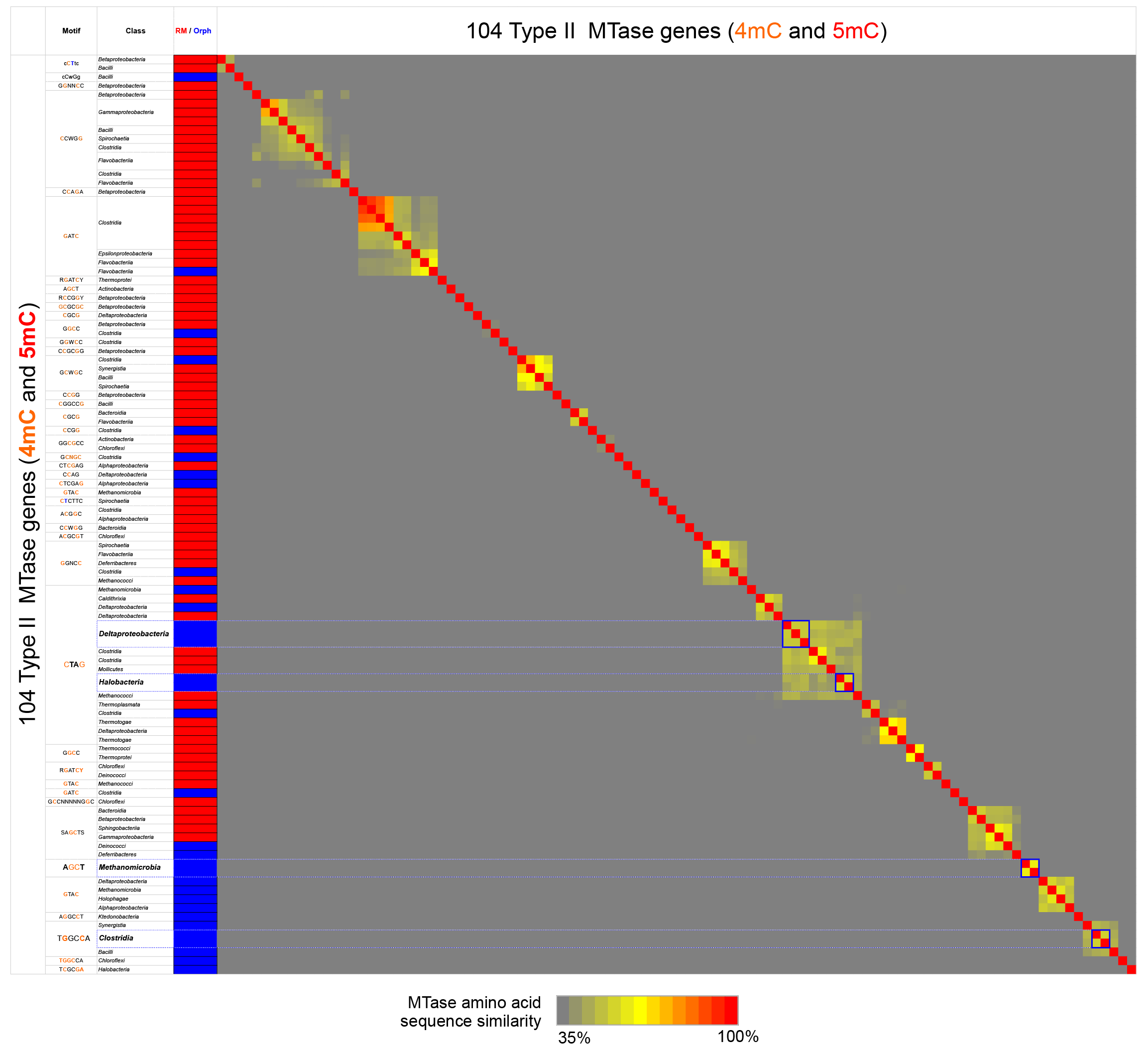

Supplement: S8 Fig — Amino acid sequence similarity was calculated for all pairs of m4C and m5C MTase with an annotated motif, and the resulting matrix of similarity scores clustered heirarchically. Each row / column corresponds to an MTase, and rows and columns are identical ordered. The matrix indicates pairwise amino acid sequence similarity from 35% (yellow) to 100% (red). Each MTase row is labeled with motif specificity, the class of organism in which the MTase is found and whether the MTase has a cognate REase (red) or is an ‘orphan’ (blue). This approach results in accurate grouping of MTases by sequence specificity. To identify candidate orphan MTase families, we manually identified sub-clusters of orphan MTases with identical or closely related specificities from taxonomically related organisms. Candidate families are indicated by blue outer lines in the matrix plot. (TIF) [file pgen.1005854.s008.tif]

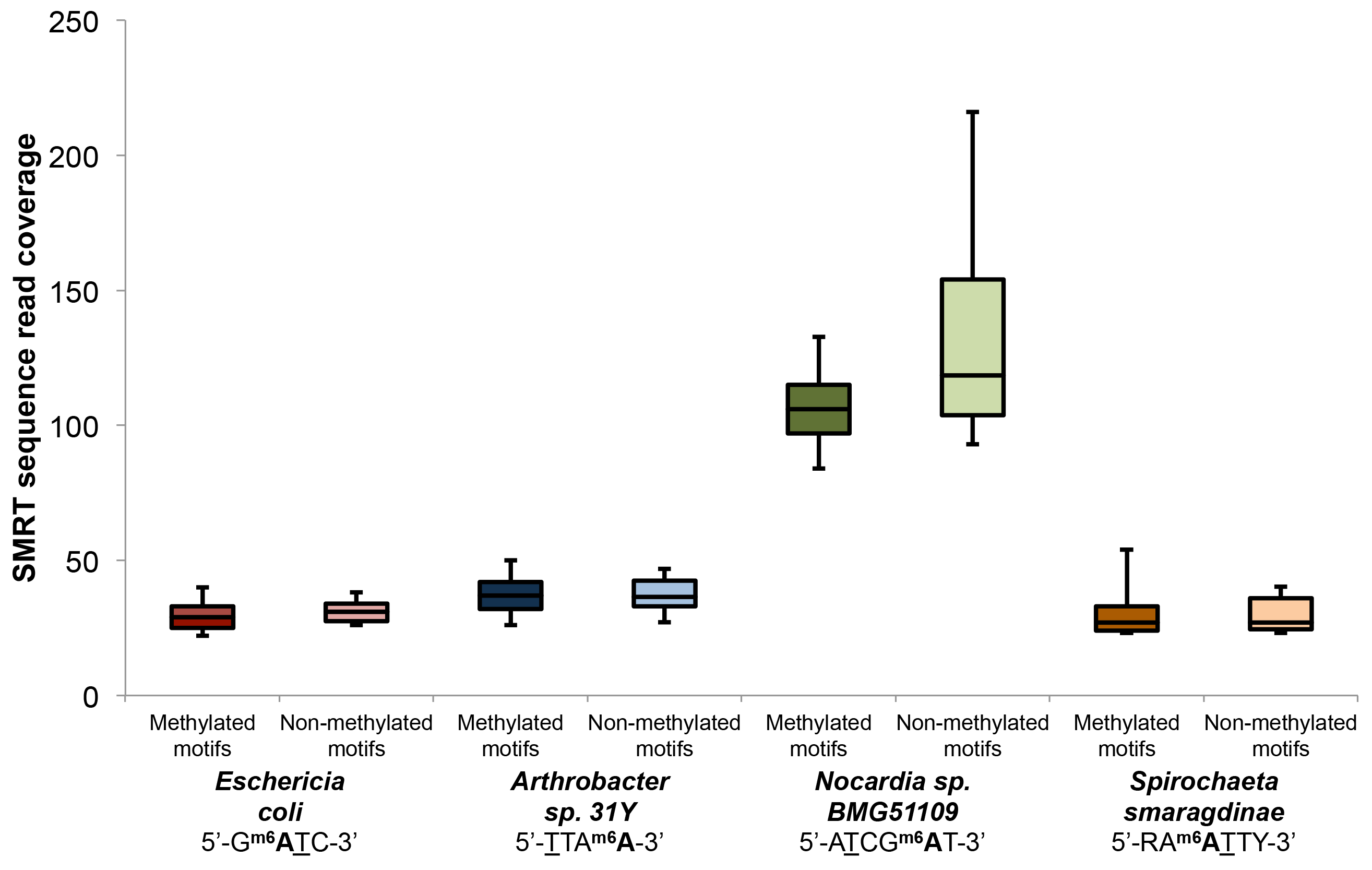

Supplement: S9 Fig — Plots indicate median, 25–75 percentile (boxes) and 5–95 percentile (whiskers) SMRT sequence coverage at methylated and unmethylated motifs, targeted by four different orphan MTases. In all cases, coverage at unmethylated sites is equivalent to or slightly greater than that at methylated sites, suggesting that the unmethylated sites associated with orphan MTases are not simply a consequence of low sequence coverage. (TIF) [file pgen.1005854.s009.tif]

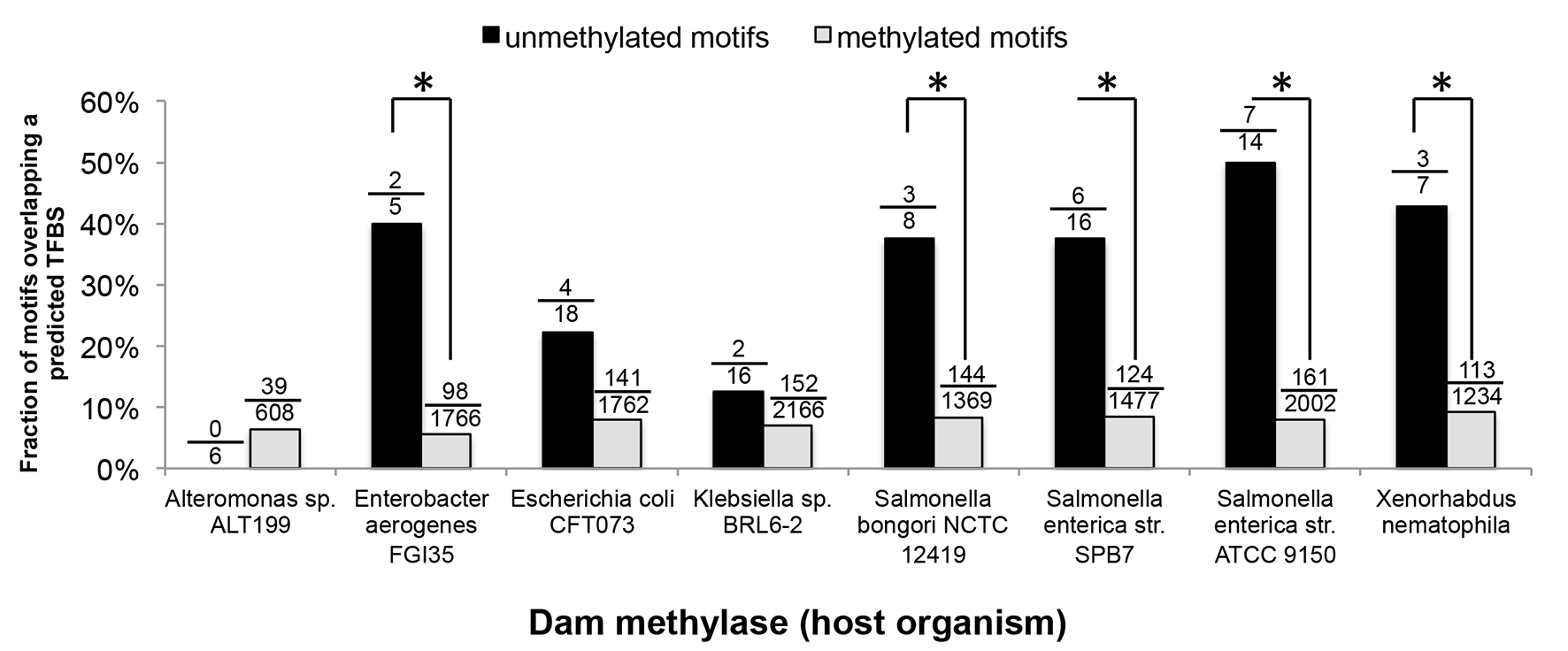

Supplement: S10 Fig — For each gammaproteobacteria with at least 5 unmethylated dam motif sites, we calculated overlap between GATC motifs and predicted transcription factor binding sites (TFBSs) in the same genome. Black bars represent proportion of unmethylated GATC motifs overlapping a TFBS. For comparison, gray bars indicate proportion of methylated GATC motifs overlapping a TFBS. The number of overlapping and non-overlapping GATC motifs is shown above each bar. In many organisms, unmethylated motifs overlap a TFBS significantly more frequently than do methylated motifs (*, p<0.05, fishers exact test). (TIF) [file pgen.1005854.s010.tif]

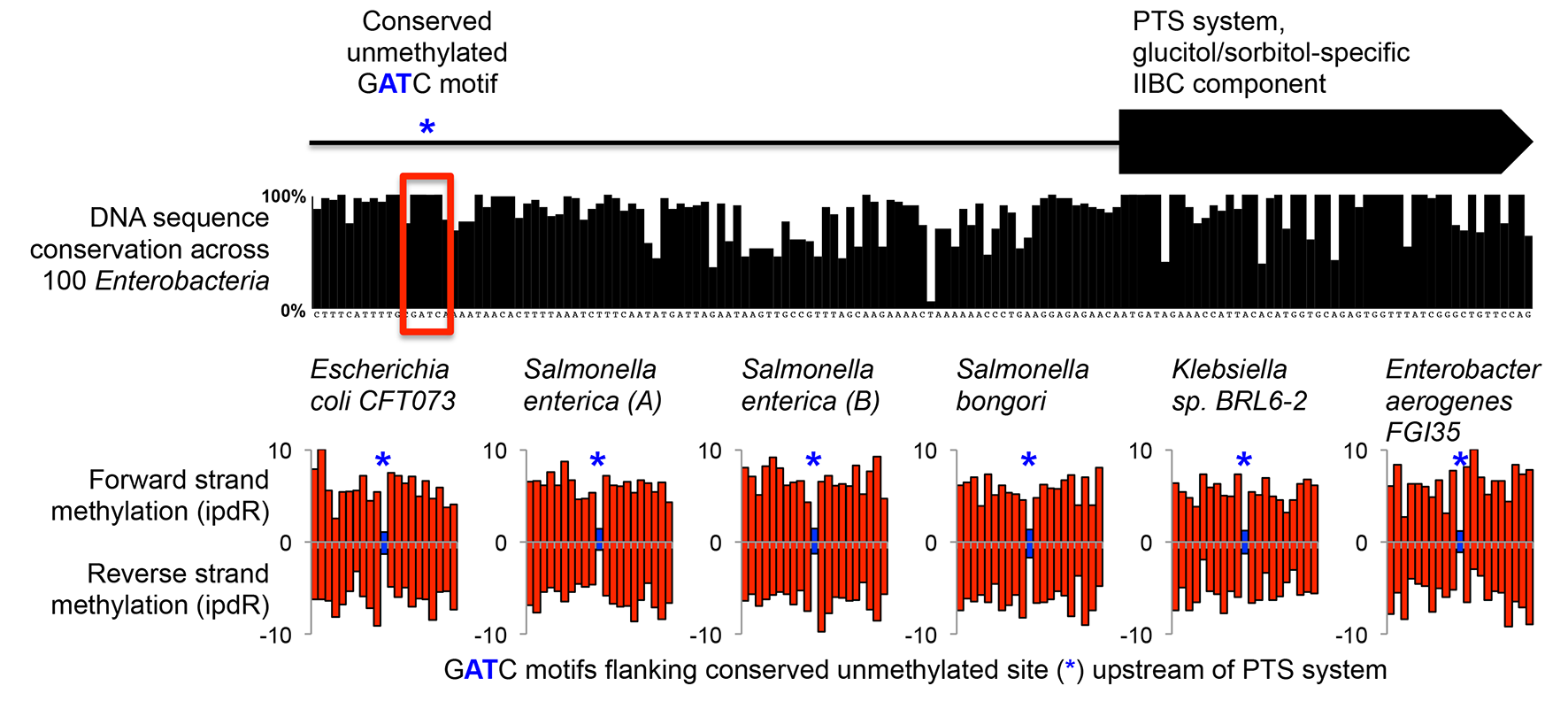

Supplement: S11 Fig — Upper panel shows a multiple sequence alignment of 100 Enterobacteria species to a 100bp sequence upstream of the PTS system in E. coli. Blue asterisk indicates the location of a conserved undermethylated site observed in 6 enterobacteria species in this study. Bar charts show the extent of DNA methylation (inter-pulse duration ratio) at the candidate regulatory unmethylated site (blue) and, for comparison, at the ten immediately flanking upstream and downstream GATC motifs (red). (TIF) [file pgen.1005854.s011.tif]
