# Supplementary material for: The Epigenomic Landscape of Prokaryotes
Source: PLoS Genet. 2016 Feb 12;12(2):e1005854. doi: 10.1371/journal.pgen.1005854 (PMC4752239; doi:10.1371/journal.pgen.1005854)
Supplement: S2 Table — Organisms without detected modification were from across the sampled taxa. In many cases the absence of methylation correlates with the absence of MTase genes in the genome. In other cases, MTases are present but not detected by SMRTsequencing. Sequencing coverage was on average higher for this set of samples, than for the study as a whole. (DOCX) [file pgen.1005854.s013.docx]

| **Organism** | **Phylum** | **Class** | **MTase in genome?** | **Type IV REase in genome?** | **SMRT sequence coverage** |
| --- | --- | --- | --- | --- | --- |
| *Acidobacterium sp. PMMR2* | Acidobacteria | Acidobacteriia | Yes | Yes | 193 |
| *Desulfurobacterium sp. TC5-1* | Aquificae | Aquificae | Yes | No | 158 |
| *Exiguobacterium acetylicum DSM 20416* | Firmicutes | Bacilli | No | Yes | 150 |
| *Lactococcus lactis lactis 511* | Firmicutes | Bacilli | Yes | No | 168 |
| *Methanothermus fervidus V24S, DSM 2088* | Euryarchaeota | Methanobacteria | No | No | 252 |
| *Methylotenera sp. 73s* | Proteobacteria | Betaproteobacteria | No | Yes | 173 |
| *Methylotenera versatilis 79* | Proteobacteria | Betaproteobacteria | No | Yes | 78 |
| *Pediococcus acidilactici AGR20* | Firmicutes | Bacilli | No | Yes | 235 |
| *Pedobacter heparinus HIM 762-3, DSM 2366* | Bacteroidetes | Sphingobacteriia | Yes | No | 45 |
| *Persephonella lauensis KM09_Lau8* | Aquificae | Aquificae | No | Yes | 169 |
| *Robinsoniella sp. KNHs210* | Firmicutes | Clostridia | Yes | Yes | 88 |
| *Sebaldella termitidis ATCC 33386* | Fusobacteria | Fusobacteriia | Yes | Yes | 113 |
| *Staphylococcus epidermidis AG42* | Firmicutes | Bacilli | No | Yes | 161 |
| *Teredinibacter sp. 1162T.S.0a.05* | Proteobacteria | Gammaproteobacteria | Yes | No | 56 |
| *Thiomonas sp. FB-6, DSM 25805* | Proteobacteria | Betaproteobacteria | No | Yes | 68 |
