# Supplementary material for: The Epigenomic Landscape of Prokaryotes
Source: PLoS Genet. 2016 Feb 12;12(2):e1005854. doi: 10.1371/journal.pgen.1005854 (PMC4752239; doi:10.1371/journal.pgen.1005854)
Supplement: S1 Text — (DOCX) [file pgen.1005854.s017.docx]

**S1 Appendix. Descriptions of Type I and Type III orphan MTases**

There are five apparent orphan type I MTases (systems that comprise an S and MTase subunit, but no adjacent REase, Fig 1D). These are:

**M.SauTCHI** and **M.SauTCHII** (*Staphylococcus aureus*). Both MTases completely modify their target sites in the genome (acAnnnnnnrTgg and AggnnnnngaT respectively). Many Staphylococcal strains sequenced to date have similar arrangements of either one complete Type I system (R+M+S genes) in one location and a second set of M+S genes in a separate location, or even just the S+M genes in one location and the R gene in a separate location. When tested it seems that the R gene product is simply shared between the two sets of genes (1). In this organism there is a solitary R gene, SauTCHORF208P, located 230 Kb away from M.SauTCHII that could pair with either of the MS genes.

**M.Lsp48ORFDP** (*Leeuwenhoekiella* *sp.* Hel_I_48). This system completely methylates its target sites in the genome (aAcnnnnncTca). There are other Type I RM systems in this genome, raising the possibility that the REase is shared between systems as in *Staphylococci*.

**M.CthIV** (*Clostridium thermocellum* ATCC 27405). This appears to be a solitary methylase / specificity subunit. The REase cannot be detected and there is no other Type I system in the genome. The target site (cncAnnnnnnTtc) is completely methylated.

**M.CmaLM2III** (*Clostridium mangenotii* LM2) This appears to be a solitary methylase / specificity subunit. The REase cannot be detected and there is no other Type I system in the genome. This is the one case of a Type I RM system that does not completely methylate its target site in the genome (ggAnnnnnvTac). There are 38 unmethylated motifs, but there no obvious bias in their distribution in the genome that would suggest a putative function.

There are four apparent Type III orphan MTases (Fig 1D).

**M.Cos21659ORFAP (***Caldicoprobacter oshimai* DSM 21659**)**

**M.VbaLP2AORFHP (***Verrucomicrobia bacterium* LP2A, tccCc**)**

**M.Aci16581I** (*Aminiphilus circumscriptus* DSM 16581, aggAg)

**M.TdeIV** (*Treponema denticola ATCC 35405*, ctaAt)

We are unable to reliably identify unmethylated target sites for Type III MTases. There is considerable variability in IPD scores which may have various technical or biological origins, and makes identification of under-methylated sites challenging. For Type I and Type II MTases, we took advantage of the fact that they recognize palindromic or bipartite repeats, and thus there are two measurements for each motif instance. Since Type III (And 2G) MTases only methylate a single strand of the genome the equivalent analysis is not possible. While there is no obvious feature of these systems that would indicate function,, there is mounting evidence that Type III methylases have important regulatory roles in phase variation (2-4).

References for appendix S1

1. Ershova AS, Karyagina AS, Vasiliev MO, Lyashchuk AM, Lunin VG, Spirin SA, et al. Solitary restriction endonucleases in prokaryotic genomes. Nucleic acids research. 2012;40(20):10107-15.

2. De Bolle X, Bayliss CD, Field D, van de Ven T, Saunders NJ, Hood DW, et al. The length of a tetranucleotide repeat tract in Haemophilus influenzae determines the phase variation rate of a gene with homology to type III DNA methyltransferases. Molecular microbiology. 2000;35(1):211-22.

3. de Vries N, Duinsbergen D, Kuipers EJ, Pot RG, Wiesenekker P, Penn CW, et al. Transcriptional phase variation of a type III restriction-modification system in Helicobacter pylori. Journal of bacteriology. 2002;184(23):6615-23.

4. Fox KL, Srikhanta YN, Jennings MP. Phase variable type III restriction-modification systems of host-adapted bacterial pathogens. Molecular microbiology. 2007;65(6):1375-9.
